# Supplementary material for: HIP (HPA-screening in pregnancy) study: protocol of a nationwide, prospective and observational study to assess incidence and natural history of fetal/neonatal alloimmune thrombocytopenia and identifying pregnancies at risk
Source: BMJ Open. 2020 Jul 20;10(7):e034071. doi: 10.1136/bmjopen-2019-034071 (PMC7375633; doi:10.1136/bmjopen-2019-034071)
Supplement: Supplementary data [file bmjopen-2019-034071supp001.pdf]

HIP-study

In the Netherlands all pregnant women are screened for antibodies against **red blood cells** as these antibodies can lead to anemia or jaundice in the newborn. In the blood of pregnant women, antibodies against **platelets** may also be present, these platelet antibodies can lead to bleeding problems in the newborn.

Can we prevent bleeding problems caused by antibodies against platelets? To answer this question we first need to know the following:

- How often are **antibodies** against platelets present in the blood of pregnant women?
- If these antibodies are present how often do they cause bleeding problems in newborn babies?
- Which newborns are at high risk of suffering from bleeding problems?
- Can we predict bleeding problems by testing the blood of pregnant women?
- How can we prevent these **bleeding problems** in newborn babies?

The HIP-study can provide answers to these questions. To answer all these questions , we need the help of midwives, gynaecologists and pregnant women. By agreeing to participate, you can make an important contribution to the HIP-study.

More information

For further information, please check
www.HIPstudie.nl
Or scan this QR-code to watch our informative video.

Please do not hesitate to consult your obstetric caregiver with any further questions you may have.
Or send an email to: **info@HIPstudie.nl**

Principal investigator:
drs. Dian Winkelhorst, medical doctor and researcher
Independent medical doctor:
Dr. M. Sueters, gynecologist, LUMC

Research study on platelet antibodies in pregnancy

HIP STUDY
HPA-screening In Pregnancy

Sanquin
LUMC
Leids Universitair Medisch Centrum

Why do you receive this flyer?

In the first trimester of your pregnancy, your red blood cell type was tested. The results showed that your red blood cells are RhD or Rhc negative. All RhD and Rhc negative pregnant women in the Netherlands are tested again at 27 weeks gestation by Sanquin in Amsterdam. We would like to perform an extra test, for the HIP-study, with the remainder of this blood sample. Here we ask your consent to perform this extra test.

What does this mean for you?

Participation is completely voluntary and will have no further consequences for the care you receive during your pregnancy. If you participate, no additional actions are necessary. No extra blood is drawn nor will you be personally contacted. You and your baby will be unaware of participation.

It is important to know that there is no personal gain in participating. However, your participation will contribute to improving knowledge on diseases caused by antibodies against platelets and will help to improve treatment during pregnancy in the future.

Which tests and actions are part of the HIP-study?

If you consent to participate in the HIP study, we will perform an extra test on the blood sample that has been already drawn. With this test we will determine the blood type of your platelets. This blood type is called **HPA-1a**. If you don't have this blood type, you are HPA-1a negative (1 in 50 women). If you are HPA-1a negative, we will store the tested blood sample. We will also store the blood sample from a small number of HPA-1a positive women as part of the so called 'control-group'.
After the expected delivery date all stored blood samples will be tested for antibodies against platelets . A member of the study team will contact your obstetric care giver to enquire on the health of your child during the first hours-days of life. All results and data will be anonymized and saved in a secured database. Results will not be reported to you or your obstetric care giver. The study results cannot be requested by third parties.

Participate?

Your obstetric care giver will ask you if you are willing to participate in the HIP-study.
Your answer will be indicated on the HIP-study special check box on the blood collection form.

Background information

Our blood contains billions of cells. For example: red blood cells, white blood cells and **platelets**. All these cells express characteristics that are called blood types. Our body can produce **antibodies** against these blood types. During pregnancy a pregnant women can produce antibodies against the blood type of her child. Sometimes these antibodies are able to destroy the blood cells of the child. This can lead to disease and the need to start timely treatment.

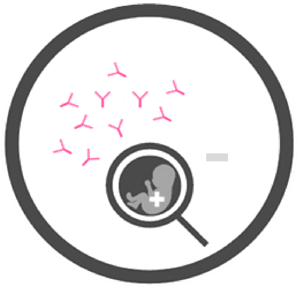

*'**HIP**' in **HIP**-study stands for:*
***H**PA-screening **I**n **P**regnancy*

***HPA** is an abbreviation for a blood type on platelets and means: Human Platelet Antigen.*
