# Supplementary material for: HIP (HPA-screening in pregnancy) study: protocol of a nationwide, prospective and observational study to assess incidence and natural history of fetal/neonatal alloimmune thrombocytopenia and identifying pregnancies at risk
Source: BMJ Open. 2020 Jul 20;10(7):e034071. doi: 10.1136/bmjopen-2019-034071 (PMC7375633; doi:10.1136/bmjopen-2019-034071)
Supplement: Author's manuscript [file bmjopen-2019-034071.draft_revisions.pdf]

BMJ Open is committed to open peer review. As part of this commitment we make the peer review history of every article we publish publicly available.

When an article is published we post the peer reviewers' comments and the authors' responses online. We also post the versions of the paper that were used during peer review. These are the versions that the peer review comments apply to.

The versions of the paper that follow are the versions that were submitted during the peer review process. They are not the versions of record or the final published versions. They should not be cited or distributed as the published version of this manuscript.

BMJ Open is an open access journal and the full, final, typeset and author-corrected version of record of the manuscript is available on our site with no access controls, subscription charges or pay-per-view fees (http://bmjopen.bmj.com).

If you have any questions on BMJ Open's open peer review process please email info.bmjopen@bmj.com

BMJ Open

HIP-study (HPA-screening In Pregnancy): Protocol of a nationwide, prospective and observational study to assess incidence and natural history of fetal/neonatal alloimmune thrombocytopenia and identifying pregnancies at risk

Journal:	*BMJ Open*
Manuscript ID	bmjopen-2019-034071
Article Type:	Protocol
Date Submitted by the Author:	04-Sep-2019
Complete List of Authors:	Winkelhorst, Dian; Leiden University Medical Center, Obstetrics; Sanquin, Department of Experimental Immunohematology, Amsterdam<br>Vos, Thijs; Leiden University Medical Center, Pediatrics; Sanquin, Department of Experimental Immunohematology, Amsterdam<br>Kamphuis, Marije M.; Onze Lieve Vrouwe Gasthuis, Obstetrics and Gynaecology<br>Porcelijn, Leendert; Sanquin Blood Supply Foundation, Immunohaematology Diagnostics<br>Lopriore, Enrico; Leiden University Medical Center, Pediatrics<br>Oepkes, Dick; Leiden University Medical Center, Obstetrics<br>van der Schoot, C. Ellen; Sanquin, Department of Experimental Immunohematology, Amsterdam and Landsteiner Laboratory, Academic Medical Center, University of Amsterdam<br>de Haas, Masja; Sanquin, Department of Immunohaematology Diagnostics, Amsterdam; Leiden University Medical Center, Immunohaematology and Blood Transfusion
Keywords:	Fetal medicine < OBSTETRICS, Prenatal diagnosis < OBSTETRICS, NEONATOLOGY

SCHOLARONE™
Manuscripts

I, the Submitting Author has the right to grant and does grant on behalf of all authors of the Work (as defined in the below author licence), an exclusive licence and/or a non-exclusive licence for contributions from authors who are: i) UK Crown employees; ii) where BMJ has agreed a CC-BY licence shall apply, and/or iii) in accordance with the terms applicable for US Federal Government officers or employees acting as part of their official duties; on a worldwide, perpetual, irrevocable, royalty-free basis to BMJ Publishing Group Ltd ("BMJ") its licensees and where the relevant Journal is co-owned by BMJ to the co-owners of the Journal, to publish the Work in this journal and any other BMJ products and to exploit all rights, as set out in our licence.

The Submitting Author accepts and understands that any supply made under these terms is made by BMJ to the Submitting Author unless you are acting as an employee on behalf of your employer or a postgraduate student of an affiliated institution which is paying any applicable article publishing charge ("APC") for Open Access articles. Where the Submitting Author wishes to make the Work available on an Open Access basis (and intends to pay the relevant APC), the terms of reuse of such Open Access shall be governed by a Creative Commons licence – details of these licences and which Creative Commons licence will apply to this Work are set out in our licence referred to above.

Other than as permitted in any relevant BMJ Author's Self Archiving Policies, I confirm this Work has not been accepted for publication elsewhere, is not being considered for publication elsewhere and does not duplicate material already published. I confirm all authors consent to publication of this Work and authorise the granting of this licence.

For peer review only

HIP-study (HPA-screening In Pregnancy): Protocol of a nationwide, prospective and observational study to assess incidence and natural history of fetal/neonatal alloimmune thrombocytopenia and identifying pregnancies at risk

Dian Winkelhorst[1,2], Thijs W. de Vos[1,3], Marije M. Kamphuis[4], Leendert Porcelijn[5], Enrico Lopriore[3], Dick Oepkes[2], C. Ellen van der Schoot[1]*, Masja de Haas[5,6]*

* equal contribution

[1]Department Experimental Immunohaematology, Sanquin Reserach, Amsterdam.
[2]Department of Obstetrics, Leiden University Medical Centre, Leiden.
[3]Division of Neonatology, Department of Paediatrics, Leiden University Medical Centre, Leiden.
[4]Department of Obstetrics and Gynaecology, Onze Lieve Vrouwen Gasthuis, Amsterdam.
[5]Department Immunohaematology Diagnostics, Sanquin Diagnostic Services, Amsterdam,
[6]Department of Immunohaematology and Blood Transfusion, Leiden University Medical Centre, Leiden.

Corresponding author:
Masja de Haas
Clustermanager Diagnostiek, Q224
Plesmanlaan 125
1066CX Amsterdam
m.dehaas@sanquin.nl
020-5123934

Email-addresses of authors:
d.winkelhorst@sanquin.nl, t.w.de_vos@lumc.nl, m.m.kamphuis@olvg.nl, l.porcelijn@sanquin.nl, e.lopriore@lumc.nl, d.oepkes@lumc.nl, e.vanderschoot@sanquin.nl, m.dehaas@sanquin.nl

For peer review only - http://bmjopen.bmj.com/site/about/guidelines.xhtml

Abstract

Introduction. Fetal and neonatal alloimmune thrombocytopenia (FNAIT) may lead to fetal or neonatal brain damage or perinatal death. Maternal alloantibodies, targeted against fetal human platelet antigens (HPAs), can result thrombocytopenia and bleeding complications. In pregnancies with known immunisation, fetal bleeding is effectively prevented using maternal intravenous immunoglobulin (IVIg) infusions. Without population-based screening, immunisation is only detected after birth of an affected infant. Affected cases that might have been prevented, when timely identified through population-based screening. Implementation is hampered by lacking knowledge on incidence, natural history and identification of pregnancies at high-risk of bleeding. We designed a study aimed to obtain this missing knowledge.

Methods and analysis. The HIP-study (HPA-screening In Pregnancy) is a nationwide, prospective and observational cohort study, aimed to assess incidence and natural history of FNAIT, as well as identifying pregnancies at high-risk for developing bleeding complications. RhD or Rhc-negative pregnant women and therefore take part in the Dutch population-based prenatal screening program for erythrocyte immunisation, are eligible for enrolment. Serological HPA-1a typing is performed and a luminex-based multiplex assay will be performed for the detection of anti-HPA-1a antibodies. Results will not be communicated to patients or caregivers. Clinical data of HPA-1a negative women and a HPA-1a positive control group will be collected after birth. Samples of HPA-1a immunised pregnancies with and without signs of bleeding will be compared to identify parameters for identification of pregnancies at high-risk for bleeding complications.

Ethics and dissemination. Ethical approval for this study has been obtained from the Committee of Medical Ethics (CME) from the LUMC (P16.002). Study enrolment began in March 2017. All pregnant women have to give informed consent for testing according to the complete protocol. Results of the study will be disseminated through congresses and publication in relevant peer-reviewed journals.

Trial registration number: NCT04067375

For peer review only

For peer review only - http://bmjopen.bmj.com/site/about/guidelines.xhtml

Article summary

Strengths and limitations of this study

- The HPA-screening In Pregnancy (HIP) study is the first prospective and completely non-interventional screening study with a large cohort that enables assessing the true natural history of fetal and neonatal alloimmune thrombocytopenia (FNAIT).
- The unique infrastructure in the Netherlands with one national referral laboratory for FNAIT (Sanquin, Amsterdam) collaborating with the national fetal therapy centre (LUMC, Leiden) will result in complete data and focus on both laboratory and clinical parameters.
- A limitation of the study is that we rely on the clinical judgement of bleeding tendency after birth, and do not obtain cord blood platelet counts or perform routine neonatal cerebral ultrasounds. Therefore, we may still underestimate disease prevalence due to subclinical cases.

Keywords: prenatal diagnosis, neonatal alloimmune thrombocytopenia, human platelet antigens, thrombocytopenia.

For peer review only

For peer review only - http://bmjopen.bmj.com/site/about/guidelines.xhtml

Introduction

Fetal and neonatal alloimmune thrombocytopenia (FNAIT) is the most frequent cause of severe thrombocytopenia in term born infants.[1, 2] FNAIT is caused by the production of maternal alloantibodies against the paternally derived, fetal human platelet antigens (HPAs). Clinical consequences can vary from an asymptomatic thrombocytopenia to minor skin haemorrhage, such as hematoma or petechiae, or ultimately severe internal organ and intracranial haemorrhage (ICH).[3, 4] Bleeding complications that, in subsequent pregnancies can be effectively prevented by weekly administration of intravenous immunoglobulins (IVIg) to the mother.[5] The vast majority of cases with (severe) clinical consequences are caused by maternal alloantibodies targeted against fetal HPA-1a.[6-8] FNAIT is considered to be the platelet counterpart of haemolytic disease of the fetus and the new-born (HDFN) because of their similar pathophysiologic fundaments. In this comparison, HPA-1a, that causes 90% of the ICH caused by FNAIT, is regarded to be the equivalent of RhD of the red blood cell (RBC) in HDFN.[8] Important differences, however, exist as well. First, whereas RhD is only expressed on red blood cells, the HPA-1a epitope expressed on platelets is also present on the membrane of endothelial cells and syncytiotrophoblast cells.[9, 10] Second, whereas RhD is mainly a problem of second or subsequent incompatible pregnancies, more than half of the severe cases of HPA-1a-mediated FNAIT already occur in first-born children.[4, 11] For decades, the possibility of prevention of FNAIT by population-based screening for HPA-1a is discussed, in analogy to the RhD prophylaxis and erythrocyte immunisation screening.[12-14]

Careful evaluation of the feasibility, benefits and harms and cost effectiveness of a possible FNAIT screening program showed that knowledge is missing on different aspects of the disease, needed before the introduction of a screening program. First, despite a couple of large prospective cohort studies, no data exist on the natural history of the disease. Most of the large prospective, screening studies performed, were not only observational, but included some kind of intervention, thereby making it impossible to draw any firm conclusion on the natural history of FNAIT.[15-19] Further, more accurate estimates of incidence and prevalence of the disease in the Dutch population need to be known. One of the most important differences, making it hard to implement a program similar to the antenatal screening program for erythrocyte immunisation, is the lack of tools to identify pregnancies at high risk for developing bleeding complications. Detecting HPA-1a negative women and further HPA-1a alloimmunised pregnancies can be easily done. When alloimmunisation is detected in HDFN several parameters, laboratory as well as clinical, are available to assess disease severity and to predict which cases would benefit from treatment. For example, RBC alloantibody titre and functional assays such as an antibody-dependent cellular cytotoxicity assay can be performed, followed in pre-selected cases by estimation of fetal anaemia by Doppler-based assessment of flow velocity in the middle cerebral artery of the fetus. In this way, high risk cases are identified that most likely benefit from fetal blood sampling (FBS), followed by an intrauterine transfusion.[20] Treating all HPA-alloimmunised pregnancies with IVIg would lead to a considerable and undesirable overtreatment. So, identification of HPA-alloimmunised pregnancies at high risk for disease, like in HDFN, would be preferable as well. FBS to determine fetal platelet count and if necessary administer intrauterine platelet transfusion, can be performed in these pregnancies as well. However, in potentially thrombocytopenic fetuses this is a risky procedure with a high rate of associated complications. Unfortunately, no non-invasive laboratory or clinical diagnostic tests to select HPA-alloimmunised pregnancies that would benefit from treatment are applicable in a clinical setting.

For peer review only - http://bmjopen.bmj.com/site/about/guidelines.xhtml

To obtain information necessary to judge the effectiveness and feasibility of a potential population-based screening, we designed the HIP (HPA-screening In Pregnancy) study. With the HIP-study we aim to collect data on the incidence of HPA-1a alloimmunisation and clinically relevant FNAIT in the Netherlands. The study will be completely observational. This way we will be able to conclude on the natural history of FNAIT. Ultimately, by comparing test characteristics of blood samples from pregnancies with and without clinical manifestations of bleeding we aim to develop on or more diagnostic tools, allowing more effective and personalised management by selecting pregnancies at high risk for bleeding complications that have the highest chance to benefit from antenatal preventive treatment with IVIg. This would not only be desirable in current management of FNAIT but especially in potential future screening setting.

For peer review only

Methods and analysis

Study objectives

The primary objective of this study is to determine incidence of HPA-1a alloimmunisation and the incidence of clinically relevant HPA-1a-induced FNAIT in the Netherlands. Clinically relevant FNAIT will be defined as minor bleeding (hematoma, bruising, petechiae or small visceral bleeding) and severe bleeding (ICH or internal organ haemorrhage) with the presence of an anti-HPA-1a alloantibody. Additionally, as secondary objective, we aim to collect a set of blood samples that can contribute to the development of a risk assessment model to be used as a diagnostic tool enabling the identification of alloimmunised pregnancies that are at high risk of developing bleeding complications.

Study design

The HIP-study is a nationwide prospective and observational cohort study, conducted in all settings of obstetric care in the Netherlands, for a period of two and a half years.

Patient and public involvement

In 2008, the ministry of Health, Welfare and Sport (In Dutch: Ministerie van VWS) gave instructions to investigate preventive interventions for 27 significant health problems that could be cost-effective. As a result the National Institute for Public Health and the Environment (in Dutch: RIVM) published a report stating that antenatal screening for FNAIT would be cost-saving, but they advised that more knowledge on natural history of the disease and treatment of detected cases should be obtained to support possible implementation of screening.[21] Also, the RIVM was involved in the design of the study. There was no further involvement of patients or public in the recruitment or the conduct of the study.

Study population

For logistic purposes, RhD or Rhc negative pregnant women were selected for enrolment in the HIP-study. As part the Dutch prenatal screening program for infectious disease and erythrocyte immunisation (in Dutch: PSIE), these women are offered a free of charge red cell antibody screening and/or fetal RHD typing at 27 weeks' gestation. For this, nine ml ethylenediamine tetra-acetic acid (EDTA) anticoagulated blood is drawn by their midwife or at certified, local laboratories all over the Netherlands (n = ±90) and transported to Sanquin laboratory in Amsterdam by regular surface mail or private courier service. The program has a voluntary participation grade of 99%.[22, 23] With approval of the RIVM, that organises this population screening program, left-over material can be used for the HIP-study for HPA-1a typing and stored for further antibody testing after informed consent.

Inclusion criteria

Prior to enrolment, participants have to fulfil these following criteria:

- Pregnant women participating in the currently implemented prenatal screening program for erythrocyte immunisation and who are typed RhD or Rhc negative.
- Ability to make an informed decision on participating in the population screening program as well as in the HIP-study.

Exclusion criteria

For peer review only - http://bmjopen.bmj.com/site/about/guidelines.xhtml

- Cases with insufficient material to perform HPA-1a typing by enzyme-linked immunosorbent assay (ELISA)
- Cases with known HPA-1a alloimmunisation

For peer review only

For peer review only - http://bmjopen.bmj.com/site/about/guidelines.xhtml

Participating centres

All obstetric care centres, hospitals, midwifery practices as well as general practices that provide obstetric care, in the Netherlands are able to enrol pregnant women to participate in the HIP-study. In order to ensure that obstetric caregivers were equipped to inform and counsel pregnant women, communicatory symposia were organised at six locations all over the Netherlands. Additionally, an informational leaflet was produced in different languages (Dutch and English on paper; Spanish, Arabic, Turkish and Polish digitally available; supplemental material). Two informational videos were made informing on FNAIT as well as the HIP-study. Lastly, a website was created containing news and information about the HIP-study (www.HIPstudie.nl).

Study outcomes

The main study parameters / primary endpoints are:

- Incidence of HPA-1a negativity in the RhD or Rhc-negative pregnant population in the Netherlands
- Incidence of HPA-1a alloantibodies in the tested population
- Incidence of clinically relevant HPA-1a-mediated FNAIT; classified into as mild or severe FNAIT
    - Severe FNAIT
        - ICH
        - internal organ haemorrhage
    - Mild FNAIT
        - Neonatal bleeding signs other than ICH or internal organ haemorrhage: hematoma, bruising, petechiae, purpura, mucosal or visceral bleeding
        - Thrombocytopenia for which treatment was administered (platelet transfusion or IVIg) or for which clinical observation was performed

Our secondary study parameters / endpoints are:

- Neonatal treatment for thrombocytopenia: platelet transfusion (with random-donor platelets versus compatible platelets), IVIg, red blood cell transfusion
- Neonatal morbidity: infection, hours/days in hospital (NICU versus Medium Care), need for additional treatment, congenital abnormalities, other causes causing increased bleeding tendency
- Neonatal laboratory findings: platelet count, haemoglobin, CRP

HIP-Study procedure

As part of the prenatal screening program for erythrocyte immunisation, an EDTA tube of blood of RhD and Rhc negative pregnant women will be sent in to Sanquin at 27 weeks' gestation. These women are eligible for enrolment in the HIP-study and will be informed about the study and asked for consent by their obstetric care givers. This consent or decline of participation is added to the regular laboratory request form for the 27th week assessment, that is already sent to Sanquin with each tube of blood. No additional blood will be drawn for the HIP-study. Once the tubes of blood are sent to Sanquin the consent is either received digitally or on paper, depending on the route and location (various hospitals, midwifery practices and local laboratories).

For peer review only - http://bmjopen.bmj.com/site/about/guidelines.xhtml

The procedures that are performed after consent and enrolment in the HIP-study can be divided into four separate phases, depending on the time in and after pregnancy (Figure I).

Phase I. After regular screening, authorisation and correspondence of the results for the prenatal screening program for erythrocyte immunisation, the tubes are made available for the HIP-study. For the HIP-study the platelet containing plasma of the stored blood tubes is serologically typed for HPA-1a, using a sandwich ELISA.[24] In short, 20 µL of plasma containing platelets will be automatically pipetted into microtiter plates that have been coated with a monoclonal antibody CLBthromb/1 (C17) directed against glycoprotein IIIa, at a concentration of 3 µg/mL to capture all platelets from the plasma. Then HRP-conjugated B2G1, an antibody targeting HPA-1a, will be added and plates will be centrifuged and incubated for 45 minutes. Lastly, after washing of the plates, HRP-substrate solution will be added for 15 minutes and after stopping of this reaction the reactions will be quantified using an ELISA reader ((Biochrom Anthos, Cambridge, United Kingdom). This HPA-1a ELISA was specifically designed for the HIP-study, thus for quick and high-throughput screening. All samples with an ELISA value below a defined optic density (OD) are called HPA-1a negative. The HPA-1a typing result is supported with an allelic discrimination polymerase chain reaction (PCR) assay. Plasma and buffy-coat of samples that are typed HPA-1a negative will be stored at -20°C, using only a study number. Additionally, for each HPA-1a negative case, material of one HPA-1a positive control will be stored simultaneously.

Because this first phase comprises serological HPA-1a typing, which is performed with fresh material, and a delay in the arrival of consent forms might exist, this phase is performed with all samples from pregnant women who did not decline participation for the HIP-study. All consecutive phases, such as antibody screening, risk-assessment development and clinical data retrieval, are solely performed in case of informed consent for the HIP-study.

For peer review only

For peer review only - http://bmjopen.bmj.com/site/about/guidelines.xhtml

Phase II. Of all samples stored with consent, obstetric care givers will be contacted to obtain clinical information. Researchers as well as obstetric care givers are blinded for HPA-1a typing results performed in phase I. An overview of the clinical parameters that will be collected is provided in figure 2. The clinical data will be stored in a secured digital database, designed by the LUMC, called ProMISe. First, study numbers of HPA-1a negative cases and HPA-1a positive controls with corresponding obstetric care givers are entered into the database. Then, for each case, ProMISe randomly generates a code. Thereafter obstetric care givers will receive a secured digital invitation to add clinical data to a digital case report form (CRF) for the cases from their practice. This secured invitation contains the initial personal data for the sample sent in for erythrocyte immunisation screening program together with the code generated by ProMISe. In the digital CRF they fill in this code and the clinical data. Clinical data is stored in ProMISe, only by anonymous study codes. This way, no personal information is being transferred or entered in our database, nor is the obstetric care giver in possession of a key that links the anonymous study number to personal information, nor does the care giver know whether their patients or clients are HPA-1a negative or positive.

Phase III. The next step is to evaluate the incidence of alloimmunisation. Of all HPA-1a negative women that gave consent for the HIP-study, we will use the stored left-over plasma to screen for HPA-1a alloantibodies. For antibody screening the Pak Lx assay, a qualitative immunoassay, will be used, according to the manufacturer's' recommendations (LIFECODES Pak Lx Assay, Immucor GTI Diagnostics, Norcross, United States of America). In short, plasma samples are incubated with reconstituted beads and for the removal of unbound antibodies, the beads are washed. Next a conjugate (anti-human immunoglobulin G antibody conjugated to phycoerythrin) is added and incubated with the sample for 30 minutes at room temperature. Lastly, the Luminex 200 instrument is used to analyse the data. The advantage of this assay is that it is quick and uses only a small amount of plasma so there will be enough left-over for further testing in phase IV.

Phase IV. Combining the results from phase II and phase III, will enable us to select cases of alloimmunisation with and without clinical manifestations of FNAIT to identify possible parameters to predict the development of (severe) bleeding complications. For this we will be testing different laboratory parameters as well as clinical parameters (Figure 2). Laboratory parameters that will be tested to assess risk at bleeding complications are: HLA-DRB3*0101 status, antibody level, Fc-core glycosylation and FcγRIII-binding index, endothelial cell binding, endothelial cell function.[25-28]

Sample size calculation

The HIP study is designed to assess the incidence of clinical relevant FNAIT in pregnant women in the Netherlands. Therefore, the incidence of ICH in HPA-1a immunised cases was compared with HPA-1a positive women. The estimated risk of ICH in immunised cases was 3%, for our power calculation we took a marge of 1% on the estimated incidence of ICH in FNAIT.[19,29] In our control group we assumed a risk on symptomatic ICH of 4.9 in 10.000 (0.05%).[30] To achieve a power of 80% at an alpha level of 5%, we calculated that a total study population of 2,400 pregnant women is needed. Within this calculation, we took into account the unequal distribution between HPA-1a positive controls and immunised cases. We considered 5% of our total study population to consist of immunised cases, which means that we need to include 120 immunised cases. Calculations were performed using logistic regression model making use of PASS 11.

For peer review only - http://bmjopen.bmj.com/site/about/guidelines.xhtml

Each year, approximately 60,000 RhD or Rhc negative pregnant women will participate in the prenatal screening program for erythrocyte immunisation and are therefore eligible for enrolment in the HIP-study. To include 120 immunised cases, we need to include 1,200 HPA-1a negative women (immunisation rate of approximately 10%).[29] Because 2.1% of the Caucasian population is HPA-1a negative, the total study population should exist of 60,000 pregnant women (Table 1). Based on previous experience with the OPZI-study and the highly positive attitude toward potential HPA-screening in pregnancy women expressed in our previous study, the expected enrolment was 50%.[31, 32] This would correspond with a study period of two years.

Statistical analysis

Clinical data will be entered into a validated data capture system, provided and designed by the LUMC. The system is protected by password and contains internal quality checks to identify inaccurate or incomplete data. Laboratory data will be entered in a separate password protected database by independent technicians, inaccessible to the researchers. Both clinical and laboratory data will be combined and further data management and analysis will be performed using SPSS (version 23.0) and Graphpad (version 8.0). An interim-analysis after 1-year will be performed.

Ethics and dissemination

Patient recruitment started in March 2017 and the study is planned to close to recruitment on the spring/summer of 2019. However, to ensure the inclusion of 1,000 – 1,500 HPA-1a negative women the inclusions period might take longer. Accurate predictions on the duration of the study will be made after interim-analysis at 1 year. Results will be published in relevant scientific journals and be disseminated in international conferences.

Discussion

FNAIT can cause severe bleeding complications in fetuses and neonates, with a high risk of associated morbidity and mortality.[33] A preventive antenatal treatment, that effectively prevents these bleeding complications from occurring, is available.[5] In current practice, this prevention is only available in pregnancies with known alloimmunisation, usually after a previously affected child. To prevent these first cases as well, timely detection by prenatal and population-based screening is necessary.

Current lack of prospective non-interventional studies providing data on natural history of the disease as well as a reliable risk assessment tool to identify alloimmunised pregnancies that are at high risk for developing bleeding, complicates the implementation of such population-based screening. The aim of the HIP-study is to gather this missing knowledge necessary to adequately evaluate the potential efficacy and feasibility of prenatal population-based screening in order to timely detect and prevent FNAIT-related complications. With the current study design and logistics, making use of the current national screening program for red blood cell immunisation with a participation grade of 99.1%, we expect our results to give an adequate representation of the Dutch population of pregnant women.

A potential limitation of this study protocol is the lack of routine determination of neonatal platelet counts. However, the goals of potential screening and prevention of FNAIT is not to prevent a low platelet count as reflected as a laboratory result, but to prevent symptomatic disease, mainly ICH, with associated morbidity caused by FNAIT. However, routine neonatal cerebral ultrasound is not

For peer review only - http://bmjopen.bmj.com/site/about/guidelines.xhtml

performed either. Therefore, cases of subclinical ICH without symptoms (such as convulsions or reduced consciousness) or additional bleeding manifestations might be missed, although in theory these might lead to developmental problems later in life. However, major ICHs detected in prospective studies that did perform routine cerebral ultrasound, were cases that were symptomatic as well.[19, 34]

Further underestimation might occur due to the fact that we will perform only a single screening for anti-HPA-1a alloantibodies, that is at 27 weeks' gestation. Immunisations that occurs later in pregnancy or after delivery will not be detected. Also, immunisations that will result in complications and termination of pregnancy or IUFD before 27 weeks' gestation will not be identified. However, in terms of assessing feasibility and cost-effectiveness of population-based screening, a slight underestimation is unquestionably preferred to an overestimation.

Overall, to our knowledge, the HIP-study will be the first study to prospectively and observationally collect data on incidence and natural history of FNAIT by including this large amount of pregnant women without performing any kind of intervention. Additionally, it will be the first study to be able to identify a unique study group, that is immunised pregnant women without disease and without intervention. This is the pre-eminent group to be used for the development of a risk-assessment platform in order to select immunised pregnancies that are at high risk to develop bleeding complications and would therefore benefit from antenatal preventive measures, such as IVIg treatment.

Acknowledgements

We want to thank the laboratory of leukocyte and platelet serology at Sanquin, Amsterdam that participated in the set-up and design of the ELISA that will be used for HPA-1a typing. Also, we want to thank the National Institute for Public Health and the Environment (in Dutch: RIVM) for their collaboration and the possibility to make use of the logistic of the current prenatal screening program for erythrocyte immunisation.

Author's contributions: All the Authors (DW, TdV, MMK, LP, EL, DO, CEvdS and MdH) participated in conceiving the study and participated in its design. DW, TdV and MdH were responsible for the coordination and promotion of the study and are responsible for the statistical analysis. MMK, LP, EL, DO and CEvdS participated in study coordination. DW and TdV led the drafting of the manuscript. LP, EL and MdH participated in drafting the manuscript. All authors approved the final manuscript.

Funding statement: This study was supported by Landsteiner Foundation for Blood Transfusion Research (LSBR) , grant number 1440.

Competing interest: None of the authors have a competing interests to declare.

Word count: 3227 words

For peer review only - http://bmjopen.bmj.com/site/about/guidelines.xhtml

Tables and figures

Table 1 – Estimated cases in HIP-study

	%	Incidence	Cases in the Netherlands *Total pregnancies n = 170,000*	Cases during study period *Total included n = 60,000**
HPA-1a negative	2.1	1 : 50	3,570	1,260
HPA-1a antibodies	10	1 : 400	428	126
Severe FNAIT	30	1 : 1,300	129	36
ICH	10 – 30	1 : 12,500	13	3-4

* Assuming 50% enrolment of the 60,000 RhD/Rhc negative women each year, for two years
FNAIT, fetal and neonatal alloimmune thrombocytopenia; HIP, HPA-screening in pregnancy; HPA, human platelet antigen; ICH, intracranial haemorrhage.

For peer review only

Figure legends:

Figure I – Schedule of selection, enrolment and tests in the HIP-study
HPA, human platelet antigen; PSIE, prenatal screening of infectious diseases and erythrocyte immunisation; RhD, rhesus D; Rhc, rhesus c.

Figure 2 – Clinical parameters

For peer review only - http://bmjopen.bmj.com/site/about/guidelines.xhtml

References

1 Burrows RF and Kelton JG. Thrombocytopenia at delivery: a prospective survey of 6715 deliveries. *Am J Obstet Gynecol* 1990; **162**(3):731-734.

2 Dreyfus M, Kaplan C, Verdy E, et al. Frequency of immune thrombocytopenia in newborns: a prospective study. Immune Thrombocytopenia Working Group. *Blood* 1997; **89**(12):4402-4406.

3 Winkelhorst D, Kamphuis MM, de Kloet LC, et al. Severe bleeding complications other than intracranial hemorrhage in neonatal alloimmune thrombocytopenia: a case series and review of the literature. *Transfusion* 2016.

4 Tiller H, Kamphuis MM, Flodmark O, et al. Fetal intracranial haemorrhages caused by fetal and neonatal alloimmune thrombocytopenia: an observational cohort study of 43 cases from an international multicentre registry. *BMJ Open* 2013; **3**(3).

5 Winkelhorst D, Murphy MF, Greinacher A, et al. Antenatal management in fetal and neonatal alloimmune thrombocytopenia: a systematic review. *Blood* 2017; **129**(11):1538-1547.

6 Mueller-Eckhardt C, Kiefel V, Grubert A, et al. 348 cases of suspected neonatal alloimmune thrombocytopenia. *Lancet* 1989; **1**(8634):363-366.

7 Newman PJ, Derbes RS and Aster RH. The human platelet alloantigens, PlA1 and PlA2, are associated with a leucine33/proline33 amino acid polymorphism in membrane glycoprotein IIIa, and are distinguishable by DNA typing. *J Clin Invest* 1989; **83**(5):1778-1781.

8 Davoren A, Curtis BR, Aster RH and McFarland JG. Human platelet antigen-specific alloantibodies implicated in 1162 cases of neonatal alloimmune thrombocytopenia. *Transfusion* 2004; **44**(8):1220-1225.

9 Campbell S, Swann HR, Seif MW, Kimber SJ and Aplin JD. Cell adhesion molecules on the oocyte and preimplantation human embryo. *Hum Reprod* 1995; **10**(6):1571-1578.

10 Sajid M and Stouffer GA. The role of alpha(v)beta3 integrins in vascular healing. *Thromb Haemost* 2002; **87**(2):187-193.

11 de Haas M, Thurik FF, Koelewijn JM and van der Schoot CE. Haemolytic disease of the fetus and newborn. *Vox Sang* 2015; **109**(2):99-113.

12 Husebekk A, Killie MK, Kjeldsen-Kragh J and Skogen B. Is it time to implement HPA-1 screening in pregnancy? *Curr Opin Hematol* 2009; **16**(6):497-502.

13 Kjeldsen-Kragh J, Ni H and Skogen B. Towards a prophylactic treatment of HPA-related foetal and neonatal alloimmune thrombocytopenia. *Curr Opin Hematol* 2012; **19**(6):469-474.

14 Murphy MF and Williamson LM. Antenatal screening for fetomaternal alloimmune thrombocytopenia: an evaluation using the criteria of the UK National Screening Committee. *Br J Haematol* 2000; **111**(3):726-732.

15 Blanchette VS, Chen L, de Friedberg ZS, et al. Alloimmunization to the PlA1 platelet antigen: results of a prospective study. *Br J Haematol* 1990; **74**(2):209-215.

16 Durand-Zaleski I, Schlegel N, Blum-Boisgard C, et al. Screening primiparous women and newborns for fetal/neonatal alloimmune thrombocytopenia: a prospective comparison of effectiveness and costs. Immune Thrombocytopenia Working Group. *Am J Perinatol* 1996; **13**(7):423-431.

17 Davoren A, McParland P, Crowley J, et al. Antenatal screening for human platelet antigen-1a: results of a prospective study at a large maternity hospital in Ireland. *BJOG* 2003; **110**(5):492-496.

18 Maslanka K, Guz K and Zupanska B. Antenatal screening of unselected pregnant women for HPA-1a antigen, antibody and alloimmune thrombocytopenia. *Vox Sang* 2003; **85**(4):326-327.

19 Kjeldsen-Kragh J, Killie MK, Tomter G, et al. A screening and intervention program aimed to reduce mortality and serious morbidity associated with severe neonatal alloimmune thrombocytopenia. *Blood* 2007; **110**(3):833-839.

20 Zwiers C, van Kamp I, Oepkes D and Lopriore E. Intrauterine transfusion and non-invasive treatment options for hemolytic disease of the fetus and newborn - review on current management and outcome. *Expert Rev Hematol* 2017; **10**(4):337-344.

21 van Gils PFT, L.; Hamberg-van Reenen, H.H.; van den Berg, M.: Kosteneffectiviteit van preventie, Rijksinstituut voor Volksgezondheid en Milieu (RIVM). 2009,

22 Van der Linden A.M.A. SY, Oomen P., Vos K.: Prenatale Screening Infectieziekten en Erytrocytenimmunisatie (PSIE): Procesmonitor 2016, TNO/RIVM, 2018,

23 van der Ploeg K, Schönbeck, Y., Oomen, P., Vos, K.: Prenatale Screening Infectieziekten en Erytrocytenimmunisatie (PSIE): Procesmonitor 2016, 2018,

24 Winkelhorst D, Porcelijn L, Muizelaar E, et al. Fast and low-cost direct ELISA for high-throughput serological HPA-1a typing. *Transfusion* 2019.

25 Kapur R, Kustiawan I, Vestrheim A, et al. A prominent lack of IgG1-Fc fucosylation of platelet alloantibodies in pregnancy. *Blood* 2014; **123**(4):471-480.

26 Kapur R, Heitink-Polle KM, Porcelijn L, et al. C-reactive protein enhances IgG-mediated phagocyte responses and thrombocytopenia. *Blood* 2015; **125**(11):1793-1802.

27 Sonneveld ME, Natunen S, Sainio S, et al. Glycosylation pattern of anti-platelet IgG is stable during pregnancy and predicts clinical outcome in alloimmune thrombocytopenia. *Br J Haematol* 2016.

28 L'Abbe D, Tremblay L, Filion M, et al. Alloimmunization to platelet antigen HPA-1a (PIA1) is strongly associated with both HLA-DRB3*0101 and HLA-DQB1*0201. *Hum Immunol* 1992; **34**(2):107-114.

29 Kamphuis MM, Paridaans N, Porcelijn L, et al. Screening in pregnancy for fetal or neonatal alloimmune thrombocytopenia: systematic review. *BJOG* 2010; **117**(11):1335-1343.

30 Gupta SN, Kechli AM and Kanamalla US. Intracranial hemorrhage in term newborns: management and outcomes. *Pediatr Neurol* 2009; **40**(1):1-12.

31 Winkelhorst D, Loeff RM, van den Akker-Van Marle ME, de Haas M and Oepkes D. Women's attitude towards routine human platelet antigen-screening in pregnancy. *Acta Obstet Gynecol Scand* 2017; **96**(8):991-997.

32 Koelewijn JM, Vrijkotte TG, van der Schoot CE, Bonsel GJ and de Haas M. Effect of screening for red cell antibodies, other than anti-D, to detect hemolytic disease of the fetus and newborn: a population study in the Netherlands. *Transfusion* 2008; **48**(5):941-952.

For peer review only - http://bmjopen.bmj.com/site/about/guidelines.xhtml

33 Winkelhorst D, Kamphuis MM, Steggerda SJ, et al. Perinatal Outcome and Long-Term Neurodevelopment after Intracranial Haemorrhage due to Fetal and Neonatal Alloimmune Thrombocytopenia. *Fetal Diagn Ther* 2018:1-8.
34 Williamson LM, Hackett G, Rennie J, et al. The natural history of fetomaternal alloimmunization to the platelet-specific antigen HPA-1a (PlA1, Zwa) as determined by antenatal screening. *Blood* 1998; **92**(7):2280-2287.

For peer review only

For peer review only - http://bmjopen.bmj.com/site/about/guidelines.xhtml

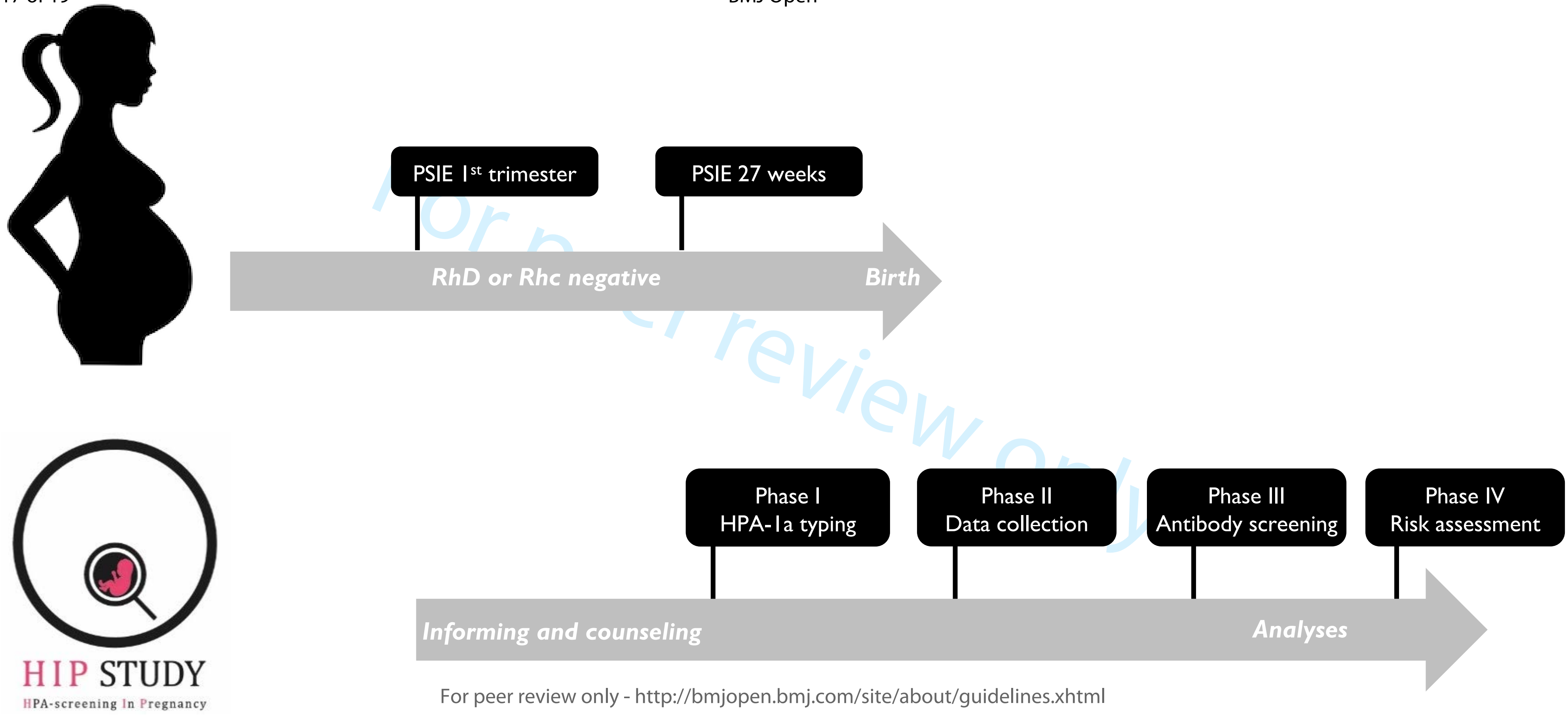

For peer review only - http://bmjopen.bmj.com/site/about/guidelines.xhtml

**Medical history:**	known with immune thrombocytopenic purpura (ITP)
**Obstetric history:**	previous pregnancies, deliveries, miscarriages (spontaneous as well as pregnancy terminations) or intra-uterine fetal demises.
**Pregnancy:**	gestational diabetes, hypertensive disorders (pregnancy-induced hypertension, pre-eclampsia), intrauterine growth restriction (IUGR).
**Perinatal:**	gestational age at delivery, mode of delivery, Apgar score, birth weight
**Neonatal:**	gender, chromosomal disorder, laboratory assessment (CRP or platelet count), consultation of pediatrician, admission to the neonatal care unit, mortality
**FNAIT-related:**	hematomas, petechiae, visceral bleeding, internal organ haemorrhage, intracranial haemorrhage, platelet count (if tested), treatment for thrombocytopenia

For peer review only - http://bmjopen.bmj.com/site/about/guidelines.xhtml

HIP-study

In the Netherlands all pregnant women are screened for antibodies against **red blood cells** as these antibodies can lead to anemia or jaundice in the newborn. In the blood of pregnant women, antibodies against **platelets** may also be present, these platelet antibodies can lead to bleeding problems in the newborn.

Can we prevent bleeding problems caused by antibodies against platelets? To answer this question we first need to know the following:

- How often are **antibodies** against platelets present in the blood of pregnant women?
- If these antibodies are present how often do they cause bleeding problems in newborn babies?
- Which newborns are at high risk of suffering from bleeding problems?
- Can we predict bleeding problems by testing the blood of pregnant women?
- How can we prevent these **bleeding problems** in newborn babies?

The HIP-study can provide answers to these questions. To answer all these questions , we need the help of midwives, gynaecologists and pregnant women. By agreeing to participate, you can make an important contribution to the HIP-study.

More information

For further information, please check
www.HIPstudie.nl
Or scan this QR-code to watch our informative video.

Please do not hesitate to consult your obstetric caregiver with any further questions you may have.
Or send an email to: **info@HIPstudie.nl**

Principal investigator:
drs. Dian Winkelhorst, medical doctor and researcher
Independent medical doctor:
Dr. M. Sueters, gynecologist, LUMC

Research study on platelet antibodies in pregnancy

HIP STUDY
HPA-screening In Pregnancy

Sanquin

LUMC
Leids Universitair Medisch Centrum

Why do you receive this flyer?

In the first trimester of your pregnancy, your red blood cell type was tested. The results showed that your red blood cells are RhD or Rhc negative. All RhD and Rhc negative pregnant women in the Netherlands are tested again at 27 weeks gestation by Sanquin in Amsterdam. We would like to perform an extra test, for the HIP-study, with the remainder of this blood sample. Here we ask your consent to perform this extra test.

What does this mean for you?

Participation is completely voluntary and will have no further consequences for the care you receive during your pregnancy. If you participate, no additional actions are necessary. No extra blood is drawn nor will you be personally contacted. You and your baby will be unaware of participation.

It is important to know that there is no personal gain in participating. However, your participation will contribute to improving knowledge on diseases caused by antibodies against platelets and will help to improve treatment during pregnancy in the future.

Which tests and actions are part of the HIP-study?

If you consent to participate in the HIP study, we will perform an extra test on the blood sample that has been already drawn. With this test we will determine the blood type of your platelets. This blood type is called **HPA-1a**. If you don't have this blood type, you are HPA-1a negative (1 in 50 women). If you are HPA-1a negative, we will store the tested blood sample. We will also store the blood sample from a small number of HPA-1a positive women as part of the so called 'control-group'.

After the expected delivery date all stored blood samples will be tested for antibodies against platelets . A member of the study team will contact your obstetric care giver to enquire on the health of your child during the first hours-days of life. All results and data will be anonymized and saved in a secured database. Results will not be reported to you or your obstetric care giver. The study results cannot be requested by third parties.

Participate?

Your obstetric care giver will ask you if you are willing to participate in the HIP-study. Your answer will be indicated on the HIP-study special check box on the blood collection form.

Background information

Our blood contains billions of cells. For example: red blood cells, white blood cells and **platelets**. All these cells express characteristics that are called blood types. Our body can produce **antibodies** against these blood types. During pregnancy a pregnant women can produce antibodies against the blood type of her child. Sometimes these antibodies are able to destroy the blood cells of the child. This can lead to disease and the need to start timely treatment.

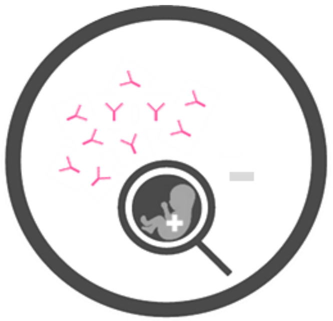

*'**HIP**' in **HIP**-study stands for:*
***H**PA-screening **I**n **P**regnancy*

***HPA** is an abbreviation for a blood type on platelets and means: Human Platelet Antigen.*

BMJ Open

HIP-study (HPA-screening In Pregnancy): Protocol of a nationwide, prospective and observational study to assess incidence and natural history of fetal/neonatal alloimmune thrombocytopenia and identifying pregnancies at risk

Journal:	*BMJ Open*
Manuscript ID	bmjopen-2019-034071.R1
Article Type:	Protocol
Date Submitted by the Author:	20-Feb-2020
Complete List of Authors:	Winkelhorst, Dian; Leiden University Medical Center, Obstetrics; Sanquin, Department of Experimental Immunohematology, Amsterdam<br>de Vos, Thijs; Leiden University Medical Center, Pediatrics; Sanquin, Department of Experimental Immunohematology, Amsterdam<br>Kamphuis, Marije M.; Onze Lieve Vrouwe Gasthuis, Obstetrics and Gynaecology<br>Porcelijn, Leendert; Sanquin Blood Supply Foundation, Immunohaematology Diagnostics<br>Lopriore, Enrico; Leiden University Medical Center, Pediatrics<br>Oepkes, Dick; Leiden University Medical Center, Obstetrics<br>van der Schoot, C. Ellen; Sanquin, Department of Experimental Immunohematology, Amsterdam and Landsteiner Laboratory, Academic Medical Center, University of Amsterdam<br>de Haas, Masja; Sanquin, Department of Immunohaematology Diagnostics, Amsterdam; Leiden University Medical Center, Immunohaematology and Blood Transfusion
**Primary Subject Heading**:	Obstetrics and gynaecology
Secondary Subject Heading:	Haematology (incl blood transfusion)
Keywords:	Fetal medicine < OBSTETRICS, Prenatal diagnosis < OBSTETRICS, NEONATOLOGY

SCHOLARONE™
Manuscripts

I, the Submitting Author has the right to grant and does grant on behalf of all authors of the Work (as defined in the below author licence), an exclusive licence and/or a non-exclusive licence for contributions from authors who are: i) UK Crown employees; ii) where BMJ has agreed a CC-BY licence shall apply, and/or iii) in accordance with the terms applicable for US Federal Government officers or employees acting as part of their official duties; on a worldwide, perpetual, irrevocable, royalty-free basis to BMJ Publishing Group Ltd ("BMJ") its licensees and where the relevant Journal is co-owned by BMJ to the co-owners of the Journal, to publish the Work in this journal and any other BMJ products and to exploit all rights, as set out in our licence.

The Submitting Author accepts and understands that any supply made under these terms is made by BMJ to the Submitting Author unless you are acting as an employee on behalf of your employer or a postgraduate student of an affiliated institution which is paying any applicable article publishing charge ("APC") for Open Access articles. Where the Submitting Author wishes to make the Work available on an Open Access basis (and intends to pay the relevant APC), the terms of reuse of such Open Access shall be governed by a Creative Commons licence – details of these licences and which Creative Commons licence will apply to this Work are set out in our licence referred to above.

Other than as permitted in any relevant BMJ Author's Self Archiving Policies, I confirm this Work has not been accepted for publication elsewhere, is not being considered for publication elsewhere and does not duplicate material already published. I confirm all authors consent to publication of this Work and authorise the granting of this licence.

For peer review only

HIP-study (HPA-screening In Pregnancy): Protocol of a nationwide, prospective and observational study to assess incidence and natural history of fetal/neonatal alloimmune thrombocytopenia and identifying pregnancies at risk

Dian Winkelhorst[1,2], Thijs W. de Vos[1,3,4], Marije M. Kamphuis[5], Leendert Porcelijn[6], Enrico Lopriore[3], Dick Oepkes[2], C. Ellen van der Schoot[1]*, Masja de Haas[4,6,7]*

* equal contribution

1. Department Experimental Immunohaematology, Sanquin Research, Amsterdam.
2. Department of Obstetrics, Leiden University Medical Centre, Leiden.
3. Division of Neonatology, Department of Paediatrics, Leiden University Medical Centre, Leiden.
4. Center of Clinical Transfusion Research, Sanquin Research, Amsterdam
5. Department of Obstetrics and Gynaecology, Onze Lieve Vrouwen Gasthuis, Amsterdam.
6. Department Immunohaematology Diagnostics, Sanquin Diagnostic Services, Amsterdam,
7. Department of Immunohaematology and Blood Transfusion, Leiden University Medical Centre, Leiden.

Corresponding author:
Masja de Haas
Laboratory Director, Immunohaematology, Q224
Plesmanlaan 125
1066CX Amsterdam
m.dehaas@sanquin.nl
020-5123934

Email-addresses of authors:
d.winkelhorst@sanquin.nl, t.w.de_vos@lumc.nl, m.m.kamphuis@olvg.nl, l.porcelijn@sanquin.nl, e.lopriore@lumc.nl, d.oepkes@lumc.nl, e.vanderschoot@sanquin.nl, m.dehaas@sanquin.nl

For peer review only

Abstract

Introduction. Fetal and neonatal alloimmune thrombocytopenia (FNAIT) may lead to severe fetal or neonatal bleeding and/or perinatal death. Maternal alloantibodies, targeted against fetal human platelet antigens (HPAs), can result thrombocytopenia and bleeding complications. In pregnancies with known immunisation, fetal bleeding can be prevented by weekly maternal intravenous immunoglobulin (IVIg) infusions. Without population-based screening, immunisation is only detected after birth of an affected infant. Affected cases that might have been prevented, when timely identified through population-based screening. Implementation is hampered by the lack of knowledge on incidence, natural history and identification of pregnancies at high-risk of bleeding. We designed a study aimed to obtain this missing knowledge.

Methods and analysis. The HIP-study (HPA-screening In Pregnancy) is a nationwide, prospective and observational cohort study, aimed to assess incidence and natural history of FNAIT, as well as identifying pregnancies at high-risk for developing bleeding complications. For logistic reasons we invite RhD or Rhc-negative pregnant women, that take part in the Dutch population-based prenatal screening program for erythrocyte immunisation, to participate in our study. Serological HPA-1a typing is performed and a luminex-based multiplex assay will be performed for the detection of anti-HPA-1a antibodies. Results will not be communicated to patients or caregivers. Clinical data of HPA-1a negative women and a HPA-1a positive control group will be collected after birth. Samples of HPA-1a immunised pregnancies with and without signs of bleeding will be compared to identify parameters for identification of pregnancies at high-risk for bleeding complications.

Ethics and dissemination. Ethical approval for this study has been obtained from the Committee of Medical Ethics (CME) from the LUMC (P16.002). Study enrolment began in March 2017. All pregnant women have to give informed consent for testing according to the complete protocol. Results of the study will be disseminated through congresses and publication in relevant peer-reviewed journals.

Trial registration number: NCT04067375

For peer review only

Article summary

Strengths and limitations of this study

- The HPA-screening In Pregnancy (HIP) study is a unique prospective and completely non-interventional screening study with a large cohort that enables assessing the true natural history of fetal and neonatal alloimmune thrombocytopenia (FNAIT).
- The unique infrastructure in the Netherlands with one national referral laboratory for FNAIT (Sanquin, Amsterdam) collaborating with the national fetal therapy centre (LUMC, Leiden) will result in complete data and focus on both laboratory and clinical parameters.
- A limitation of the study is that we rely on the clinical judgement of bleeding tendency after birth, and do not obtain cord blood platelet counts or perform routine neonatal cerebral ultrasounds. Therefore, we may still underestimate disease prevalence due to subclinical cases.

Keywords: prenatal diagnosis, neonatal alloimmune thrombocytopenia, human platelet antigens, thrombocytopenia.

Introduction

Fetal and neonatal alloimmune thrombocytopenia (FNAIT) is the most frequent cause of severe thrombocytopenia in term born infants.[1, 2] FNAIT is caused by the production of maternal alloantibodies against the paternally derived, fetal human platelet antigens (HPAs). Clinical consequences can vary from an asymptomatic thrombocytopenia to minor skin haemorrhage, such as hematoma or petechiae, or ultimately severe internal organ and intracranial haemorrhage (ICH).[3, 4] Bleeding complications that, in subsequent pregnancies can be effectively prevented by weekly administration of intravenous immunoglobulins (IVIg) to the mother.[5] The vast majority of cases with (severe) clinical consequences are caused by maternal alloantibodies targeted against fetal HPA-1a.[6-8] FNAIT is considered to be the platelet counterpart of haemolytic disease of the fetus and the new-born (HDFN) because of their similar pathophysiologic fundaments. In this comparison, HPA-1a, that causes 90% of the ICH caused by FNAIT, is regarded to be the equivalent of RhD of the red blood cell (RBC) in HDFN.[8] Important differences, however, exist as well. First, whereas RhD is only expressed on red blood cells, the HPA-1a epitope expressed on platelets is also present on the membrane of endothelial cells and syncytiotrophoblast cells.[9, 10] Second, whereas RhD is mainly a problem of second or subsequent incompatible pregnancies, more than half of the severe cases of HPA-1a-mediated FNAIT already occur in first-born children.[4, 11] For decades, the possibility of prevention of FNAIT by population-based screening for HPA-1a is discussed, in analogy to the RhD prophylaxis and erythrocyte immunisation screening.[12-14]

Careful evaluation of the feasibility, benefits and harms and cost effectiveness of a possible FNAIT screening program showed that knowledge is missing on different aspects of the disease, needed before the introduction of a screening program. First, despite a couple of large prospective cohort studies, no data exist on the natural history of the disease. Most of the large prospective, screening studies performed, were not only observational, but included some kind of intervention, thereby making it impossible to draw any firm conclusion on the natural history of FNAIT.[15-19] Further, more accurate estimates of incidence and prevalence of the disease in the Dutch population need to be known. One of the most important differences, making it hard to implement a program similar to the antenatal screening program for erythrocyte immunisation, is the lack of tools to identify pregnancies at high risk for developing bleeding complications. Detecting HPA-1a negative women and further HPA-1a alloimmunised pregnancies can be easily done. When alloimmunisation is detected in HDFN several parameters, laboratory as well as clinical, are available to assess disease severity and to predict which cases would benefit from treatment. For example, RBC alloantibody titre and functional assays such as an antibody-dependent cellular cytotoxicity assay can be performed, followed in pre-selected cases by estimation of fetal anaemia by Doppler-based assessment of flow velocity in the middle cerebral artery of the fetus. In this way, high risk cases are identified that most likely benefit from fetal blood sampling (FBS), followed by an intrauterine transfusion.[20] Treating all HPA-alloimmunised pregnancies with IVIg would lead to a considerable and undesirable overtreatment. So, identification of HPA-alloimmunised pregnancies at high risk for disease, like in HDFN, would be preferable as well. FBS to determine fetal platelet count and if necessary administer intrauterine platelet transfusion, can be performed in these pregnancies as well. However, in potentially thrombocytopenic fetuses this is a risky procedure with a high rate of associated complications. Unfortunately, no non-invasive laboratory or clinical diagnostic tests to select HPA-alloimmunised pregnancies that would benefit from treatment are applicable in a clinical setting.

To obtain information necessary to judge the effectiveness and feasibility of a potential population-based screening, we designed the HIP (HPA-screening In Pregnancy) study. With the HIP-study we aim

For peer review only - http://bmjopen.bmj.com/site/about/guidelines.xhtml

to collect data on the incidence of HPA-1a alloimmunisation and clinically relevant FNAIT in the Netherlands. The study will be completely observational. This way we will be able to conclude on the natural history of FNAIT. Ultimately, by comparing test characteristics of blood samples from pregnancies with and without clinical manifestations of bleeding we aim to develop on or more diagnostic tools, allowing more effective and personalised management by selecting pregnancies at high risk for bleeding complications that have the highest chance to benefit from antenatal preventive treatment with IVIg. This would not only be desirable in current management of FNAIT but especially in potential future screening setting.

Methods and analysis

Study objectives

The primary objective of this study is to determine incidence of HPA-1a alloimmunisation and the incidence of clinically relevant HPA-1a-induced FNAIT in the Netherlands. Clinically relevant FNAIT will be defined as minor bleeding (hematoma, bruising, petechiae or small visceral bleeding) and severe bleeding (ICH or internal organ haemorrhage) with the presence of an anti-HPA-1a alloantibody. Additionally, as secondary objective, we aim to collect a set of blood samples that can contribute to the development of a risk assessment model to be used as a diagnostic tool enabling the identification of alloimmunised pregnancies that are at high risk of developing bleeding complications.

Study design

The HIP-study is a nationwide prospective and observational cohort study, conducted in all settings of obstetric care in the Netherlands, for a period of two and a half years.

Patient and public involvement

In 2008, the ministry of Health, Welfare and Sport (In Dutch: Ministerie van VWS) gave instructions to investigate preventive interventions for 27 significant health problems that could be cost-effective. As a result the National Institute for Public Health and the Environment (in Dutch: RIVM) published a report stating that antenatal screening for FNAIT would be cost-saving, but they advised that more knowledge on natural history of the disease and treatment of detected cases should be obtained to support possible implementation of screening.[21] Also, the RIVM was involved in the design of the study. There was no further involvement of patients or public in the recruitment or the conduct of the study.

Study population

For logistic purposes, RhD or Rhc negative pregnant women were selected for enrolment in the HIP-study. As part the Dutch prenatal screening program for infectious disease and erythrocyte immunisation (in Dutch: PSIE), these women are offered a free of charge red cell antibody screening and/or fetal RHD typing at 27 weeks' gestation. For this, nine ml ethylenediamine tetra-acetic acid (EDTA) anticoagulated blood is drawn by their midwife or at certified, local laboratories all over the Netherlands (*n* = ±90) and transported to Sanquin laboratory in Amsterdam by regular surface mail or private courier service. The program has a voluntary participation grade of 99%.[22, 23] With approval of the RIVM, that organises this population screening program, left-over material can be used for the HIP-study for HPA-1a typing and stored for further antibody testing after informed consent.

For peer review only

Inclusion criteria

Prior to enrolment, participants have to fulfil these following criteria:

- Pregnant women participating in the currently implemented prenatal screening program for erythrocyte immunisation and who are typed RhD or Rhc negative.
- Ability to make an informed decision on participating in the population screening program as well as in the HIP-study.

Exclusion criteria

- Cases with insufficient material to perform HPA-1a typing by enzyme-linked immunosorbent assay (ELISA)
- Cases with known HPA-1a alloimmunisation

Participating centres

All obstetric care centres, hospitals, midwifery practices as well as general practices that provide obstetric care, in the Netherlands are able to enrol pregnant women to participate in the HIP-study. In order to ensure that obstetric caregivers were equipped to inform and counsel pregnant women, communicatory symposia were organised at six locations all over the Netherlands. Additionally, an informational leaflet was produced in different languages (Dutch and English on paper; Spanish, Arabic, Turkish and Polish digitally available; supplemental material). Two informational videos were made informing on FNAIT as well as the HIP-study. Lastly, a website was created containing news and information about the HIP-study (www.HIPstudie.nl).

Study outcomes

The main study parameters / primary endpoints are:

- Incidence of HPA-1a negativity in the RhD or Rhc-negative pregnant population in the Netherlands at 27 weeks in pregnancy
- Incidence of HPA-1a alloantibodies in the tested population at 27 weeks in pregnancy
- Incidence of clinically relevant HPA-1a-mediated FNAIT; classified into as mild or severe FNAIT
    - Severe FNAIT
        - ICH
        - internal organ haemorrhage
    - Mild FNAIT
        - Neonatal bleeding signs other than ICH or internal organ haemorrhage: hematoma, bruising, petechiae, purpura, mucosal or visceral bleeding
        - Thrombocytopenia for which treatment was administered (platelet transfusion or IVIg) or for which clinical observation was performed

Our secondary study parameters / endpoints are:

- Neonatal treatment for thrombocytopenia: platelet transfusion (with random-donor platelets versus compatible platelets), IVIg, red blood cell transfusion
- Neonatal morbidity: small for gestational age, infection, hours/days in hospital (NICU versus Medium Care), need for additional treatment, congenital abnormalities, other causes causing increased bleeding tendency
- Neonatal laboratory findings: platelet count, haemoglobin, CRP

For peer review only

HIP-Study procedure

As part of the prenatal screening program for erythrocyte immunisation, an EDTA tube of blood of RhD and Rhc negative pregnant women will be sent in to Sanquin at 27 weeks' gestation. These women are eligible for enrolment in the HIP-study and will be informed about the study and asked for consent by their obstetric care givers. This consent or decline of participation is added to the regular laboratory request form for the 27th week assessment, that is already sent to Sanquin with each tube of blood. No additional blood will be drawn for the HIP-study. Once the tubes of blood are sent to Sanquin the consent is either received digitally or on paper, depending on the route and location (various hospitals, midwifery practices and local laboratories).

The procedures that are performed after consent and enrolment in the HIP-study can be divided into four separate phases, depending on the time in and after pregnancy (Figure I).

Phase I. After regular screening, authorisation and correspondence of the results for the prenatal screening program for erythrocyte immunisation, the tubes are made available for the HIP-study. For the HIP-study the platelet containing plasma of the stored blood tubes is serologically typed for HPA-1a, using a sandwich ELISA. In short, 20 µL of plasma containing platelets will be automatically pipetted into microtiter plates that have been coated with a monoclonal antibody CLBthromb/1 (C17) directed against glycoprotein IIIa, at a concentration of 3 µg/mL to capture all platelets from the plasma. Then HRP-conjugated B2G1, an antibody targeting HPA-1a, will be added and plates will be centrifuged and incubated for 45 minutes. Lastly, after washing of the plates, HRP-substrate solution will be added for 15 minutes and after stopping of this reaction the reactions will be quantified using an ELISA reader ((Biochrom Anthos, Cambridge, United Kingdom). This HPA-1a ELISA was specifically designed for the HIP-study, thus for quick and high-throughput screening. All samples with an ELISA value below a defined optic density (OD) are called HPA-1a negative. The HPA-1a typing result is supported with an allelic discrimination polymerase chain reaction (PCR) assay. Plasma and buffy-coat of samples that are typed HPA-1a negative will be stored at -20°C, using only a study number. Additionally, for each HPA-1a negative case, material of one HPA-1a positive control will be stored simultaneously.

Because this first phase comprises serological HPA-1a typing, which is performed with fresh material, and a delay in the arrival of consent forms might exist, this phase is performed with all samples from pregnant women who did not decline participation for the HIP-study. All consecutive phases, such as antibody screening, risk-assessment development and clinical data retrieval, are solely performed in case of informed consent for the HIP-study.

Phase II. Of all samples stored with consent, obstetric care givers will be contacted to obtain clinical information. An overview of these clinical parameters is provided in figure 2. The clinical data will be stored in a secured digital database, designed by the LUMC, called ProMISe. First, study numbers of HPA-1a negative cases and HPA-1a positive controls with corresponding obstetric care givers are entered into the database. Then, for each case, ProMISe randomly generates a code. Thereafter obstetric care givers will receive a secured digital invitation to add clinical data to a digital case report form (CRF) for the cases from their practice. This secured invitation contains the initial personal data for the sample sent in for erythrocyte immunisation screening program together with the code generated by ProMISe. In the digital CRF they fill in this code and the clinical data. Clinical data is stored in ProMISe, only by anonymous study codes. This way, no personal information is being transferred or entered in our database, nor is the obstetric care giver in possession of a key that links the anonymous study number

For peer review only

to personal information, nor does the care giver know whether their patients or clients are HPA-1a negative or positive.

Phase III. The next step is to evaluate the incidence of alloimmunisation. Of all HPA-1a negative women that gave consent for the HIP-study, we will use the stored left-over plasma to screen for HPA-1a alloantibodies. For antibody screening the Pak Lx assay, a qualitative immunoassay, will be used, according to the manufacturer's' recommendations (LIFECODES Pak Lx Assay, Immucor GTI Diagnostics, Norcross, United States of America). In short, plasma samples are incubated with reconstituted beads and for the removal of unbound antibodies, the beads are washed. Next a conjugate (anti-human immunoglobulin G antibody conjugated to phycoerythrin) is added and incubated with the sample for 30 minutes at room temperature. Lastly, the Luminex 200 instrument is used to analyse the data. The advantage of this assay is that it is quick and uses only a small amount of plasma so there will be enough left-over for further testing in phase IV.

Phase IV. Combining the results from phase II and phase III, will enable us to select cases of alloimmunisation with and without clinical manifestations of FNAIT to identify possible parameters to predict the development of (severe) bleeding complications. For this we will be testing different laboratory parameters as well as clinical parameters (Figure 2). Laboratory parameters that will be tested to assess risk at bleeding complications are: HLA-DRB3*0101 status, antibody level, Fc-core glycosylation and FcγRIII-binding index, endothelial cell binding, endothelial cell function. (24-28)

Sample size calculation

The HIP study is designed to assess the incidence of clinical relevant FNAIT in pregnant women in the Netherlands. Therefore, the incidence of ICH in HPA-1a immunised cases was compared with HPA-1a positive women. The estimated risk of ICH in immunised cases was 3%, for our power calculation we took a marge of 1% on the estimated incidence of ICH in FNAIT.[(19),(29)] In our control group we assumed a risk on symptomatic ICH of 4.9 in 10.000 (0.05%).[(30)] To achieve a power of 80% at an alpha level of 5%, we calculated that a total study population of 2,400 pregnant women is needed. Within this calculation, we took into account the unequal distribution between HPA-1a positive controls and immunised cases. We considered 5% of our total study population to consist of immunised cases, which means that we need to include 120 immunised cases. Calculations were performed using logistic regression model making use of PASS 11.

Each year, approximately 60,000 RhD or Rhc negative pregnant women will participate in the prenatal screening program for erythrocyte immunisation and are therefore eligible for enrolment in the HIP-study. To include 120 immunised cases, we need to include 1,200 HPA-1a negative women (immunisation rate of approximately 10%).[(29)] Because 2.1% of the Caucasian population is HPA-1a negative, the total study population should exist of 60,000 pregnant women (Table 1). Based on previous experience with the OPZI-study and the highly positive attitude toward potential HPA-screening in pregnancy women expressed in our previous study, the expected enrolment was 50%.[(31, 32)] This would correspond with a study period of two years.

Statistical analysis

Clinical data will be entered into a validated data capture system, provided and designed by the LUMC. The system is protected by password and contains internal quality checks to identify inaccurate or incomplete data. Laboratory data will be entered in a separate password protected database by

For peer review only

independent technicians, inaccessible to the researchers. Both clinical and laboratory data will be combined and further data management and analysis will be performed using SPSS (version 23.0) and Graphpad (version 8.0). An interim-analysis after 1-year will be performed.

Ethics and dissemination

The introduction of an antenatal screening program requires a careful balance between benefit and potential harm. To investigate the true natural history of FNAIT we aimed to collect data from pregnancies without additional interventions based on screening test results, this observational non-intervention design is ethically challenging. It would be unethical to share the antibody screening results with pregnant women but withhold them from therapy, therefore antibody screening will be performed far after due date. There will be no direct beneficial effect for pregnant women participating in the HIP study, pregnant women will be informed by their caregivers about this before they give consent to our study.

Patient recruitment started in March 2017 and the study is planned to close to recruitment on the spring/summer of 2019. However, to ensure the inclusion of 1,000 – 1,500 HPA-1a negative women the inclusions period might take longer. Accurate predictions on the duration of the study will be made after interim-analysis at 1 year. Results will be published in relevant scientific journals and be disseminated in international conferences when inclusion and clinical data collection is finished.

Discussion

FNAIT can cause severe bleeding complications in fetuses and neonates, with a high risk of associated morbidity and mortality.[(33)] A preventive antenatal treatment, that effectively prevents these bleeding complications from occurring, is available.[(5)] In current practice, this prevention is only available in pregnancies with known alloimmunisation, usually after a previously affected child. To prevent these first cases as well, timely detection by prenatal and population-based screening is necessary.

Current lack of prospective non-interventional studies providing data on natural history of the disease as well as a reliable risk assessment tool to identify alloimmunised pregnancies that are at high risk for developing bleeding, complicates the implementation of such population-based screening. The aim of the HIP-study is to gather this missing knowledge necessary to adequately evaluate the potential efficacy and feasibility of prenatal population-based screening in order to timely detect and prevent FNAIT-related complications. With the current study design and logistics, making use of the current national screening program for red blood cell immunisation with a participation grade of 99.1%. We do not think that selection of RhD and Rhc negative women would influence the outcome of our study (i.e. immunization rate or bleeding symptoms). RhD and Rhc status has never been associated with platelet immunization during pregnancy and inheritance is unrelated since the RhD and Rhc genes are located on chromosome 1 and the HPA-1 allele on chromosome 17. Therefore we expect our results to give an adequate representation of the Dutch population of pregnant women.

A potential limitation of this study protocol is the lack of routine determination of neonatal platelet counts. However, the goals of potential screening and prevention of FNAIT is not to prevent a low platelet count as reflected as a laboratory result, but to prevent symptomatic disease, mainly ICH, with associated morbidity caused by FNAIT. However, routine neonatal cerebral ultrasound is not performed

For peer review only

either. Therefore, cases of subclinical ICH without symptoms (such as convulsions or reduced consciousness) or additional bleeding manifestations might be missed, although in theory these might lead to developmental problems later in life. However, major ICHs detected in prospective studies that did perform routine cerebral ultrasound, were cases that were symptomatic as well.[19, 34]

Further underestimation might occur due to the fact that we will perform only a single screening for anti-HPA-1a alloantibodies, that is at 27 weeks' gestation. Immunisations that occurs later in pregnancy or after delivery will not be detected. Also, immunisations that will result in complications and termination of pregnancy or IUFD before 27 weeks' gestation will not be identified. However, in terms of assessing feasibility and cost-effectiveness of population-based screening, a slight underestimation is unquestionably preferred to an overestimation. On the contrary we use another antibody screening method compared to earlier screening studies which is possibly more sensitive compared to the MAIPA technique (Monoclonal Antibody Immobilization of Platelet Antigens [35]) The PAK Lx assay was tested on a series with 100 cases with suspected FNAIT by our research group in 2014. In 26 of these cases anti-HPA-1a was detected by MAIPA, all cases were detected and one more by PAKLx. Overall, to our knowledge, the HIP-study will be a unique study to prospectively and observationally collect data on incidence and natural history of FNAIT by including this large amount of pregnant women without performing any kind of intervention. Additionally, it will be the first study to be able to identify a unique and unbiased study group, that is immunised pregnant women without disease and without intervention. This is the pre-eminent group to be used for the development of a risk-assessment platform in order to select immunised pregnancies that are at high risk to develop bleeding complications and would therefore benefit from antenatal preventive measures, such as IVIg treatment.

Contributorship statement

DW, DO, CES, MH designed the study, MK, LP, EL commented on the design of the HIP study; DW, TV, LP, DO, CES and MH wrote study materials and coordinated the study design, wrote and reviewed this paper.

Competing interests

The authors declare that they have no conflict of interest.

Funding

This project was funded by Landsteiner Foundation for Blood Transfusion Research (1440).

Data availability statement

Data Availability Statement was not applicable, this article does not contain any data.

For peer review only

Reference list

1. Burrows RF, Kelton JG. Thrombocytopenia at delivery: a prospective survey of 6715 deliveries. Am J Obstet Gynecol. 1990;162(3):731-4.
2. Dreyfus M, Kaplan C, Verdy E, Schlegel N, Durand-Zaleski I, Tchernia G. Frequency of immune thrombocytopenia in newborns: a prospective study. Immune Thrombocytopenia Working Group. Blood. 1997;89(12):4402-6.
3. Winkelhorst D, Kamphuis MM, de Kloet LC, Zwaginga JJ, Oepkes D, Lopriore E. Severe bleeding complications other than intracranial hemorrhage in neonatal alloimmune thrombocytopenia: a case series and review of the literature. Transfusion. 2016.
4. Tiller H, Kamphuis MM, Flodmark O, Papadogiannakis N, David AL, Sainio S, et al. Fetal intracranial haemorrhages caused by fetal and neonatal alloimmune thrombocytopenia: an observational cohort study of 43 cases from an international multicentre registry. BMJ Open. 2013;3(3).
5. Winkelhorst D, Murphy MF, Greinacher A, Shehata N, Bakchoul T, Massey E, et al. Antenatal management in fetal and neonatal alloimmune thrombocytopenia: a systematic review. Blood. 2017;129(11):1538-47.
6. Mueller-Eckhardt C, Kiefel V, Grubert A, Kroll H, Weisheit M, Schmidt S, et al. 348 cases of suspected neonatal alloimmune thrombocytopenia. Lancet. 1989;1(8634):363-6.
7. Newman PJ, Derbes RS, Aster RH. The human platelet alloantigens, PlA1 and PlA2, are associated with a leucine33/proline33 amino acid polymorphism in membrane glycoprotein IIIa, and are distinguishable by DNA typing. J Clin Invest. 1989;83(5):1778-81.
8. Davoren A, Curtis BR, Aster RH, McFarland JG. Human platelet antigen-specific alloantibodies implicated in 1162 cases of neonatal alloimmune thrombocytopenia. Transfusion. 2004;44(8):1220-5.
9. Campbell S, Swann HR, Seif MW, Kimber SJ, Aplin JD. Cell adhesion molecules on the oocyte and preimplantation human embryo. Hum Reprod. 1995;10(6):1571-8.
10. Sajid M, Stouffer GA. The role of alpha(v)beta3 integrins in vascular healing. Thromb Haemost. 2002;87(2):187-93.
11. de Haas M, Thurik FF, Koelewijn JM, van der Schoot CE. Haemolytic disease of the fetus and newborn. Vox Sang. 2015;109(2):99-113.
12. Husebekk A, Killie MK, Kjeldsen-Kragh J, Skogen B. Is it time to implement HPA-1 screening in pregnancy? Curr Opin Hematol. 2009;16(6):497-502.
13. Kjeldsen-Kragh J, Ni H, Skogen B. Towards a prophylactic treatment of HPA-related foetal and neonatal alloimmune thrombocytopenia. Curr Opin Hematol. 2012;19(6):469-74.
14. Murphy MF, Williamson LM. Antenatal screening for fetomaternal alloimmune thrombocytopenia: an evaluation using the criteria of the UK National Screening Committee. Br J Haematol. 2000;111(3):726-32.
15. Blanchette VS, Chen L, de Friedberg ZS, Hogan VA, Trudel E, Decary F. Alloimmunization to the PlA1 platelet antigen: results of a prospective study. Br J Haematol. 1990;74(2):209-15.
16. Durand-Zaleski I, Schlegel N, Blum-Boisgard C, Uzan S, Dreyfus M, Kaplan C. Screening primiparous women and newborns for fetal/neonatal alloimmune thrombocytopenia: a prospective comparison of effectiveness and costs. Immune Thrombocytopenia Working Group. Am J Perinatol. 1996;13(7):423-31.
17. Davoren A, McParland P, Crowley J, Barnes A, Kelly G, Murphy WG. Antenatal screening for human platelet antigen-1a: results of a prospective study at a large maternity hospital in Ireland. BJOG. 2003;110(5):492-6.
18. Maslanka K, Guz K, Zupanska B. Antenatal screening of unselected pregnant women for HPA-1a antigen, antibody and alloimmune thrombocytopenia. Vox Sang. 2003;85(4):326-7.
19. Kjeldsen-Kragh J, Killie MK, Tomter G, Golebiowska E, Randen I, Hauge R, et al. A screening and intervention program aimed to reduce mortality and serious morbidity associated with severe neonatal alloimmune thrombocytopenia. Blood. 2007;110(3):833-9.
20. Zwiers C, van Kamp I, Oepkes D, Lopriore E. Intrauterine transfusion and non-invasive treatment options for hemolytic disease of the fetus and newborn - review on current management and outcome. Expert Rev Hematol. 2017;10(4):337-44.
21. van Gils PFT, L.; Hamberg-van Reenen, H.H.; van den Berg, M. Kosteneffectiviteit van preventie. Rijksinstituut voor Volksgezondheid en Milieu (RIVM). 2009.
22. Van der Linden A.M.A. SY, Oomen P., Vos K. Prenatale Screening Infectieziekten en Erytrocytenimmunisatie (PSIE). TNO/RIVM; 2018.
23. van der Ploeg K, Schönbeck, Y., Oomen, P., Vos, K. Prenatale Screening Infectieziekten en Erytrocytenimmunisatie (PSIE). 2018.
24. Kapur R, Kustiawan I, Vestrheim A, Koeleman CA, Visser R, Einarsdottir HK, et al. A prominent lack of IgG1-Fc fucosylation of platelet alloantibodies in pregnancy. Blood. 2014;123(4):471-80.
25. Kapur R, Heitink-Polle KM, Porcelijn L, Bentlage AE, Bruin MC, Visser R, et al. C-reactive protein enhances IgG-mediated phagocyte responses and thrombocytopenia. Blood. 2015;125(11):1793-802.
26. Sonneveld ME, Natunen S, Sainio S, Koeleman CA, Holst S, Dekkers G, et al. Glycosylation pattern of anti-platelet IgG is stable during pregnancy and predicts clinical outcome in alloimmune thrombocytopenia. Br J Haematol. 2016.
27. L'Abbe D, Tremblay L, Filion M, Busque L, Goldman M, Decary F, et al. Alloimmunization to platelet antigen HPA-1a (PIA1) is strongly associated with both HLA-DRB3*0101 and HLA-DQB1*0201. Hum Immunol. 1992;34(2):107-14.
28. Metcalfe P, Allen D, Chapman J, Ouwehand WH. Interlaboratory variation in the detection of clinically significant alloantibodies against human platelet alloantigens. Br J Haematol. 1997;97(1):204-7.
29. Kamphuis MM, Paridaans N, Porcelijn L, De Haas M, Van Der Schoot CE, Brand A, et al. Screening in pregnancy for fetal or neonatal alloimmune thrombocytopenia: systematic review. BJOG. 2010;117(11):1335-43.
30. Gupta SN, Kechli AM, Kanamalla US. Intracranial hemorrhage in term newborns: management and outcomes. Pediatric neurology. 2009;40(1):1-12.
31. Winkelhorst D, Loeff RM, van den Akker-Van Marle ME, de Haas M, Oepkes D. Women's attitude towards routine human platelet antigen-screening in pregnancy. Acta obstetricia et gynecologica Scandinavica. 2017;96(8):991-7.
32. Koelewijn JM, Vrijkotte TG, van der Schoot CE, Bonsel GJ, de Haas M. Effect of screening for red cell antibodies, other than anti-D, to detect hemolytic disease of the fetus and newborn: a population study in the Netherlands. Transfusion. 2008;48(5):941-52.
33. Winkelhorst D, Kamphuis MM, Steggerda SJ, Rijken M, Oepkes D, Lopriore E, et al. Perinatal Outcome and Long-Term Neurodevelopment after Intracranial Haemorrhage due to Fetal and Neonatal Alloimmune Thrombocytopenia. Fetal Diagn Ther. 2018:1-8.

For peer review only

34. Williamson LM, Hackett G, Rennie J, Palmer CR, Maciver C, Hadfield R, et al. The natural history of fetomaternal alloimmunization to the platelet-specific antigen HPA-1a (PlA1, Zwa) as determined by antenatal screening. Blood. 1998;92(7):2280-7.
35. Porcelijn L, Huiskes E, Comijs-van Osselen I, Chhatta A, Rathore V, Meyers M, et al. A new bead-based human platelet antigen antibodies detection assay versus the monoclonal antibody immobilization of platelet antigens assay. Transfusion. 2014;54(6):1486-92.

Tables

Table 1 – Estimated cases in HIP-study

	%	Incidence	Cases in the Netherlands *Total pregnancies n = 170,000*	Cases during study period *Total included n = 60,000**
HPA-1a negative	2.1	1 : 50	3,570	1,260
HPA-1a antibodies	10	1 : 400	428	126
Severe FNAIT	30	1 : 1,300	129	36
ICH	10 – 30	1 : 12,500	13	3-4

* Assuming 50% enrolment of the 60,000 RhD/Rhc negative women each year, for two years
FNAIT, fetal and neonatal alloimmune thrombocytopenia; HIP, HPA-screening in pregnancy; HPA, human platelet antigen; ICH, intracranial haemorrhage.

Figures

Figure 1 – Schedule of selection, enrolment and tests in the HIP-study
HPA, human platelet antigen; PSIE, prenatal screening of infectious diseases and erythrocyte immunisation; RhD, rhesus D; Rhc, rhesus c.

Figure 2 – Clinical parameters

These clinical parameters will be collected in the HIP study.

Supplemental information

Supplemental material 1 - Patient information leaflet

For peer review only

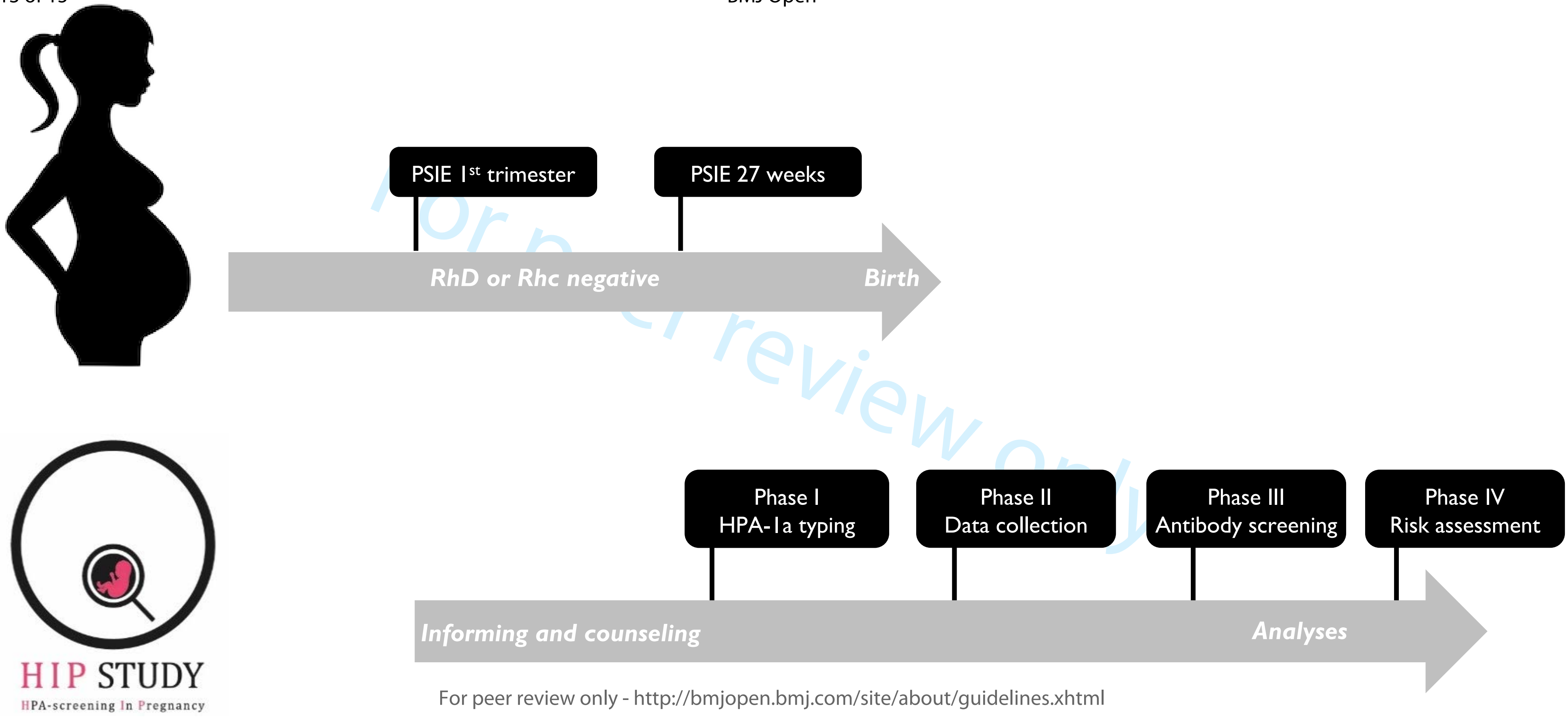

For peer review only - http://bmjopen.bmj.com/site/about/guidelines.xhtml

**Medical history:**	known with immune thrombocytopenic purpura (ITP)
**Obstetric history:**	previous pregnancies, deliveries, miscarriages (spontaneous as well as pregnancy terminations) or intra-uterine fetal demises.
**Pregnancy:**	gestational diabetes, hypertensive disorders (pregnancy-induced hypertension, pre-eclampsia), intrauterine growth restriction (IUGR).
**Perinatal:**	gestational age at delivery, mode of delivery, Apgar score, birth weight
**Neonatal:**	gender, chromosomal disorder, laboratory assessment (CRP or platelet count), consultation of pediatrician, admission to the neonatal care unit, mortality
**FNAIT-related:**	hematomas, petechiae, visceral bleeding, internal organ haemorrhage, intracranial haemorrhage, platelet count (if tested), treatment for thrombocytopenia

These clinical parameters will be collected in the HIP study.

415x190mm (96 x 96 DPI)

HIP-study

In the Netherlands all pregnant women are screened for antibodies against **red blood cells** as these antibodies can lead to anemia or jaundice in the newborn. In the blood of pregnant women, antibodies against **platelets** may also be present, these platelet antibodies can lead to bleeding problems in the newborn.

Can we prevent bleeding problems caused by antibodies against platelets? To answer this question we first need to know the following:

- How often are **antibodies** against platelets present in the blood of pregnant women?
- If these antibodies are present how often do they cause bleeding problems in newborn babies?
- Which newborns are at high risk of suffering from bleeding problems?
- Can we predict bleeding problems by testing the blood of pregnant women?
- How can we prevent these **bleeding problems** in newborn babies?

The HIP-study can provide answers to these questions. To answer all these questions , we need the help of midwives, gynaecologists and pregnant women. By agreeing to participate, you can make an important contribution to the HIP-study.

More information

For further information, please check
www.HIPstudie.nl
Or scan this QR-code to watch our informative video.

Please do not hesitate to consult your obstetric caregiver with any further questions you may have.
Or send an email to: **info@HIPstudie.nl**

Principal investigator:
drs. Dian Winkelhorst, medical doctor and researcher
Independent medical doctor:
Dr. M. Sueters, gynecologist, LUMC

Research study on platelet antibodies in pregnancy

HIP STUDY
HPA-screening In Pregnancy

Sanquin

LUMC
Leids Universitair Medisch Centrum

For peer review only - http://bmjopen.bmj.com/site/about/guidelines.xhtml

Why do you receive this flyer?

In the first trimester of your pregnancy, your red blood cell type was tested. The results showed that your red blood cells are RhD or Rhc negative. All RhD and Rhc negative pregnant women in the Netherlands are tested again at 27 weeks gestation by Sanquin in Amsterdam. We would like to perform an extra test, for the HIP-study, with the remainder of this blood sample. Here we ask your consent to perform this extra test.

What does this mean for you?

Participation is completely voluntary and will have no further consequences for the care you receive during your pregnancy. If you participate, no additional actions are necessary. No extra blood is drawn nor will you be personally contacted. You and your baby will be unaware of participation.

It is important to know that there is no personal gain in participating. However, your participation will contribute to improving knowledge on diseases caused by antibodies against platelets and will help to improve treatment during pregnancy in the future.

Which tests and actions are part of the HIP-study?

If you consent to participate in the HIP study, we will perform an extra test on the blood sample that has been already drawn. With this test we will determine the blood type of your platelets. This blood type is called **HPA-1a**. If you don't have this blood type, you are HPA-1a negative (1 in 50 women). If you are HPA-1a negative, we will store the tested blood sample. We will also store the blood sample from a small number of HPA-1a positive women as part of the so called 'control-group'.
After the expected delivery date all stored blood samples will be tested for antibodies against platelets . A member of the study team will contact your obstetric care giver to enquire on the health of your child during the first hours-days of life. All results and data will be anonymized and saved in a secured database. Results will not be reported to you or your obstetric care giver. The study results cannot be requested by third parties.

Participate?

Your obstetric care giver will ask you if you are willing to participate in the HIP-study.
Your answer will be indicated on the HIP-study special check box on the blood collection form.

Background information

Our blood contains billions of cells. For example: red blood cells, white blood cells and **platelets**. All these cells express characteristics that are called blood types. Our body can produce **antibodies** against these blood types. During pregnancy a pregnant women can produce antibodies against the blood type of her child. Sometimes these antibodies are able to destroy the blood cells of the child. This can lead to disease and the need to start timely treatment.

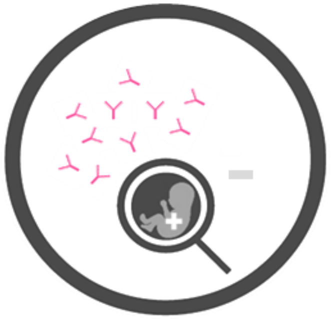

*'**HIP**' in **HIP**-study stands for:*
***H**PA-screening **I**n **P**regnancy*

***HPA** is an abbreviation for a blood type on platelets and means: Human Platelet Antigen.*

For peer review only - http://bmjopen.bmj.com/site/about/guidelines.xhtml

BMJ Open

HIP-study (HPA-screening In Pregnancy): Protocol of a nationwide, prospective and observational study to assess incidence and natural history of fetal/neonatal alloimmune thrombocytopenia and identifying pregnancies at risk

Journal:	*BMJ Open*
Manuscript ID	bmjopen-2019-034071.R2
Article Type:	Protocol
Date Submitted by the Author:	05-May-2020
Complete List of Authors:	Winkelhorst, Dian; Leiden University Medical Center, Obstetrics; Sanquin, Department of Experimental Immunohematology, Amsterdam<br>de Vos, Thijs; Leiden University Medical Center, Pediatrics; Sanquin, Department of Experimental Immunohematology, Amsterdam<br>Kamphuis, Marije M.; Onze Lieve Vrouwe Gasthuis, Obstetrics and Gynaecology<br>Porcelijn, Leendert; Sanquin Blood Supply Foundation, Immunohaematology Diagnostics<br>Lopriore, Enrico; Leiden University Medical Center, Pediatrics<br>Oepkes, Dick; Leiden University Medical Center, Obstetrics<br>van der Schoot, C. Ellen; Sanquin, Department of Experimental Immunohematology, Amsterdam and Landsteiner Laboratory, Academic Medical Center, University of Amsterdam<br>de Haas, Masja; Sanquin, Department of Immunohaematology Diagnostics, Amsterdam; Leiden University Medical Center, Immunohaematology and Blood Transfusion
**Primary Subject Heading**:	Obstetrics and gynaecology
Secondary Subject Heading:	Haematology (incl blood transfusion)
Keywords:	Fetal medicine < OBSTETRICS, Prenatal diagnosis < OBSTETRICS, NEONATOLOGY

SCHOLARONE™
Manuscripts

I, the Submitting Author has the right to grant and does grant on behalf of all authors of the Work (as defined in the below author licence), an exclusive licence and/or a non-exclusive licence for contributions from authors who are: i) UK Crown employees; ii) where BMJ has agreed a CC-BY licence shall apply, and/or iii) in accordance with the terms applicable for US Federal Government officers or employees acting as part of their official duties; on a worldwide, perpetual, irrevocable, royalty-free basis to BMJ Publishing Group Ltd ("BMJ") its licensees and where the relevant Journal is co-owned by BMJ to the co-owners of the Journal, to publish the Work in this journal and any other BMJ products and to exploit all rights, as set out in our licence.

The Submitting Author accepts and understands that any supply made under these terms is made by BMJ to the Submitting Author unless you are acting as an employee on behalf of your employer or a postgraduate student of an affiliated institution which is paying any applicable article publishing charge ("APC") for Open Access articles. Where the Submitting Author wishes to make the Work available on an Open Access basis (and intends to pay the relevant APC), the terms of reuse of such Open Access shall be governed by a Creative Commons licence – details of these licences and which Creative Commons licence will apply to this Work are set out in our licence referred to above.

Other than as permitted in any relevant BMJ Author's Self Archiving Policies, I confirm this Work has not been accepted for publication elsewhere, is not being considered for publication elsewhere and does not duplicate material already published. I confirm all authors consent to publication of this Work and authorise the granting of this licence.

For peer review only

HIP-study (HPA-screening In Pregnancy): Protocol of a nationwide, prospective and observational study to assess incidence and natural history of fetal/neonatal alloimmune thrombocytopenia and identifying pregnancies at risk

Dian Winkelhorst[1,2], Thijs W. de Vos[1,3,4], Marije M. Kamphuis[5], Leendert Porcelijn[6], Enrico Lopriore[3], Dick Oepkes[2], C. Ellen van der Schoot[6,1]*, Masja de Haas[4,,7,8]*

* equal contribution

1. Department Experimental Immunohaematology, Sanquin Research, Amsterdam.
2. Department of Obstetrics, Leiden University Medical Centre, Leiden.
3. Division of Neonatology, Department of Paediatrics, Leiden University Medical Centre, Leiden.
4. Center of Clinical Transfusion Research, Sanquin Research, Amsterdam
5. Department of Obstetrics and Gynaecology, Onze Lieve Vrouwen Gasthuis, Amsterdam.
6. Landsteiner Laboratory, Academic Medical Centre, University of Amsterdam, Amsterdam
7. Department Immunohaematology Diagnostics, Sanquin Diagnostic Services, Amsterdam.
8. Department of Immunohaematology and Blood Transfusion, Leiden University Medical Centre, Leiden.

Corresponding author:
Ellen van der Schoot
Head of the department of Experimental Immunohematology, U2
Plesmanlaan 125
1066CX Amsterdam
e.vanderschoot@sanquin.nl
020-512 3377

Email-addresses of authors:
d.winkelhorst@sanquin.nl, t.w.de_vos@lumc.nl, m.m.kamphuis@olvg.nl, l.porcelijn@sanquin.nl, e.lopriore@lumc.nl, d.oepkes@lumc.nl, e.vanderschoot@sanquin.nl, m.dehaas@sanquin.nl

For peer review only

Abstract

Introduction. Fetal and neonatal alloimmune thrombocytopenia (FNAIT) may lead to severe fetal or neonatal bleeding and/or perinatal death. Maternal alloantibodies, targeted against fetal human platelet antigens (HPAs), can result thrombocytopenia and bleeding complications. In pregnancies with known immunisation, fetal bleeding can be prevented by weekly maternal intravenous immunoglobulin (IVIg) infusions. Without population-based screening, immunisation is only detected after birth of an affected infant. Affected cases that might have been prevented, when timely identified through population-based screening. Implementation is hampered by the lack of knowledge on incidence, natural history and identification of pregnancies at high-risk of bleeding. We designed a study aimed to obtain this missing knowledge.

Methods and analysis. The HIP-study (HPA-screening In Pregnancy) is a nationwide, prospective and observational cohort study, aimed to assess incidence and natural history of FNAIT, as well as identifying pregnancies at high-risk for developing bleeding complications. For logistic reasons we invite RhD or Rhc-negative pregnant women, that take part in the Dutch population-based prenatal screening program for erythrocyte immunisation, to participate in our study. Serological HPA-1a typing is performed and a luminex-based multiplex assay will be performed for the detection of anti-HPA-1a antibodies. Results will not be communicated to patients or caregivers. Clinical data of HPA-1a negative women and an HPA-1a positive control group will be collected after birth. Samples of HPA-1a immunised pregnancies with and without signs of bleeding will be compared to identify parameters for identification of pregnancies at high-risk for bleeding complications.

Ethics and dissemination. Ethical approval for this study has been obtained from the Medical Ethical Committee Leiden-The Hague-Delft (METC-LDD) (P16.002). Study enrolment began in March 2017. All pregnant women have to give informed consent for testing according to the protocol. Results of the study will be disseminated through congresses and publication in relevant peer-reviewed journals.

Trial registration number: NCT04067375

For peer review only

Article summary

Strengths and limitations of this study

- The HPA-screening In Pregnancy (HIP) study is a unique prospective and completely non-interventional screening study with a large cohort that enables assessing the true natural history of fetal and neonatal alloimmune thrombocytopenia (FNAIT).
- The unique infrastructure in the Netherlands with one national referral laboratory for FNAIT (Sanquin, Amsterdam) collaborating with the national fetal therapy centre (LUMC, Leiden) will result in complete data and focus on both laboratory and clinical parameters.
- A limitation of the study is that we rely on the clinical judgement of bleeding tendency after birth, and do not obtain cord blood platelet counts or perform routine neonatal cerebral ultrasounds. Therefore, we may still underestimate disease prevalence due to subclinical cases.

Keywords: prenatal diagnosis, neonatal alloimmune thrombocytopenia, human platelet antigens, thrombocytopenia.

Introduction

Fetal and neonatal alloimmune thrombocytopenia (FNAIT) is the most frequent cause of severe thrombocytopenia in term born infants.[1, 2] FNAIT is caused by the production of maternal alloantibodies against the paternally derived, fetal human platelet antigens (HPAs). Clinical consequences can vary from an asymptomatic thrombocytopenia to minor skin haemorrhage, such as haematoma or petechiae, or ultimately severe internal organ and intracranial haemorrhage (ICH).[3, 4] Bleeding complications that, in subsequent pregnancies can be effectively prevented by weekly administration of intravenous immunoglobulins (IVIg) to the mother.[5] The vast majority of cases with (severe) clinical consequences are caused by maternal alloantibodies targeted against fetal HPA-1a.[6-8] FNAIT is considered to be the platelet counterpart of haemolytic disease of the fetus and the newborn (HDFN) because of their similar pathophysiologic fundaments. In this comparison, HPA-1a, that causes 90% of the ICH caused by FNAIT, is regarded to be the equivalent of RhD of the red blood cell (RBC) in HDFN.[8] Important differences, however, exist as well. First, whereas RhD is only expressed on red blood cells, the HPA-1a epitope expressed on platelets is also present on the membrane of endothelial cells and syncytiotrophoblast cells.[9, 10] Second, whereas RhD is mainly a problem of second or subsequent incompatible pregnancies, more than half of the severe cases of HPA-1a-mediated FNAIT already occur in firstborn children.[4, 11] For decades, the possibility of prevention of FNAIT by population-based screening for HPA-1a is discussed, in analogy to the RhD prophylaxis and erythrocyte immunisation screening.[12-14]

Careful evaluation of the feasibility, benefits, harms and cost effectiveness of a possible FNAIT screening program showed that knowledge is missing on different aspects of the disease. First, despite a couple of large prospective cohort studies, no data exist on the natural history of the disease. Most of the large prospective, screening studies performed, were not only observational, but included some kind of intervention, thereby making it impossible to draw any firm conclusion on the natural history of FNAIT.[15-19] Further, more accurate estimates of incidence and prevalence of the disease in the Dutch population need to be known. One of the most important differences, making it hard to implement a program similar to the antenatal screening program for erythrocyte immunisation, is the lack of tools to identify pregnancies at high risk for developing bleeding complications. Detecting HPA-1a negative women and further HPA-1a alloimmunised pregnancies can be done easily. When alloimmunisation is detected in HDFN several parameters, laboratory as well as clinical, are available to assess disease severity and to predict which cases would benefit from treatment. For example, RBC alloantibody titre and functional assays such as an antibody-dependent cellular cytotoxicity assay can be performed, followed in pre-selected cases by estimation of fetal anaemia by Doppler-based assessment of flow velocity in the middle cerebral artery of the fetus. In this way, high risk cases are identified that most likely benefit from fetal blood sampling (FBS), followed by an intrauterine transfusion.[20] Treating all HPA-alloimmunised pregnancies with IVIg would lead to a considerable and undesirable overtreatment. So, identification of HPA-alloimmunised pregnancies at high risk for disease, like in HDFN, would be preferable as well. FBS to determine fetal platelet count and if necessary administer intrauterine platelet transfusion, can be performed in these pregnancies as well. However, in potentially thrombocytopenic fetuses this is a risky procedure with a high rate of associated complications.[5] Unfortunately, no non-invasive laboratory or clinical diagnostic tests to select HPA-alloimmunised pregnancies that would benefit from treatment are applicable in a clinical setting.

To obtain information necessary to judge the effectiveness and feasibility of a potential population-based screening, we designed the HIP (HPA-screening In Pregnancy) study. With the HIP-study we aim to collect data on the incidence of HPA-1a alloimmunisation and clinically relevant FNAIT in the

For peer review only

Netherlands. The study will be completely observational. This way we will be able to conclude on the natural history of FNAIT. Ultimately, by comparing test characteristics of blood samples from pregnancies with and without clinical manifestations of bleeding we aim to develop one or more diagnostic tools, allowing more effective and personalised management by selecting pregnancies at high risk for bleeding complications that have the highest chance to benefit from antenatal preventive treatment with IVIg. This would not only be desirable in current management of FNAIT but especially in potential future screening setting.

Methods and analysis

Study objectives

The primary objective of this study is to determine incidence of HPA-1a alloimmunisation and the incidence of clinically relevant HPA-1a-induced FNAIT in the Netherlands. Clinically relevant FNAIT will be defined as minor bleeding (haematoma, bruising, petechiae or small visceral bleeding) and severe bleeding (ICH or internal organ haemorrhage) with the presence of an anti-HPA-1a alloantibody. Additionally, as secondary objective, we aim to collect a set of blood samples that can contribute to the development of a risk assessment model to be used as a diagnostic tool enabling the identification of alloimmunised pregnancies that are at high risk of developing bleeding complications.

Study design

The HIP-study is a nationwide prospective and observational cohort study, conducted in all settings of obstetric care in the Netherlands, for a period of two and a half years.

Patient and public involvement

In 2008, the ministry of Health, Welfare and Sport (in Dutch: Ministerie van VWS) gave instructions to investigate preventive interventions for 27 significant health problems that could be cost-effective. As a result the National Institute for Public Health and the Environment (in Dutch: RIVM) published a report stating that antenatal screening for FNAIT would be cost-saving, but they advised that more knowledge on natural history of the disease and treatment of detected cases should be obtained to support possible implementation of screening.[21] Also, the RIVM was involved in the design of the study. There was no further involvement of patients or public in the recruitment or the conduct of the study.

Study population

For logistic purposes, RhD or Rhc negative pregnant women were selected for enrolment in the HIP-study. As part of the Dutch prenatal screening program for infectious disease and erythrocyte immunisation (in Dutch: PSIE), these women are offered a free of charge red cell antibody screening and/or fetal RHD typing at 27 weeks' gestation. For this, nine ml ethylenediamine tetra-acetic acid (EDTA) anticoagulated blood is drawn by their midwife or at certified, local laboratories all over the Netherlands (*n* = ±90) and transported to the Sanquin laboratory in Amsterdam by regular surface mail or private courier service. The program has a voluntary participation grade of 99%.[22, 23] With approval of the RIVM, that organises this population screening program, left-over material can be used for the HIP-study for HPA-1a typing and stored for further antibody testing after informed consent.

For peer review only

Inclusion criteria

Prior to enrolment, participants have to fulfil these following criteria:

- Pregnant women participating in the currently implemented prenatal screening program for erythrocyte immunisation and who are typed RhD or Rhc negative.
- Ability to make an informed decision on participating in the population screening program as well as in the HIP-study.

Exclusion criteria

- Cases with insufficient material to perform HPA-1a typing by enzyme-linked immunosorbent assay (ELISA).
- Cases with known HPA-1a alloimmunisation.

Participating centres

All obstetric care centres, hospitals, midwifery practices as well as general practices that provide obstetric care, in the Netherlands are able to enrol pregnant women to participate in the HIP-study. In order to ensure that obstetric caregivers were equipped to inform and counsel pregnant women, communicatory symposia were organised at six locations all over the Netherlands. Additionally, an informational leaflet was produced in different languages (Dutch and English on paper; Spanish, Arabic, Turkish and Polish digitally available; supplemental material). Two informational videos were made informing on FNAIT as well as the HIP-study. Lastly, a website was created containing news and information about the HIP-study (www.HIPstudie.nl).

Study outcomes

The main study parameters / primary endpoints are:

- Incidence of HPA-1a negativity in the RhD or Rhc-negative pregnant population in the Netherlands at 27 weeks of pregnancy
- Incidence of HPA-1a alloantibodies in the tested population at 27 weeks of pregnancy
- Incidence of clinically relevant HPA-1a-mediated FNAIT; classified as mild or severe FNAIT
    - Severe FNAIT
        - ICH
        - Internal organ haemorrhage
    - Mild FNAIT
        - Neonatal bleeding signs other than ICH or internal organ haemorrhage: haematoma, bruising, petechiae, purpura, mucosal or visceral bleeding
        - Thrombocytopenia for which treatment was administered (platelet transfusion or IVIg) or for which clinical observation was performed

Our secondary study parameters / endpoints are:

- Neonatal treatment for thrombocytopenia: platelet transfusion (with random-donor platelets versus compatible platelets), IVIg, red blood cell transfusion
- Neonatal morbidity: small for gestational age, infection, hours/days in hospital (NICU versus Medium Care), need for additional treatment, congenital abnormalities, other causes causing increased bleeding tendency
- Neonatal laboratory findings: platelet count, haemoglobin, CRP

For peer review only

HIP-Study procedure

As part of the prenatal screening program for erythrocyte immunisation, an EDTA tube of blood of RhD and Rhc negative pregnant women will be sent to Sanquin at 27 weeks' gestation. These women are eligible for enrolment in the HIP-study and will be informed about the study and asked for consent by their obstetric caregivers. This consent or decline of participation is added to the regular laboratory request form for the 27th week assessment, that is already sent to Sanquin with each tube of blood. No additional blood will be drawn for the HIP-study. Once the tubes of blood are sent to Sanquin the consent is either received digital or on paper, depending on the route and location (various hospitals, midwifery practices and local laboratories).

The procedures that are performed after consent and enrolment in the HIP-study can be divided into four separate phases, depending on the time in and after pregnancy (Figure I).

Phase I. After regular screening, authorisation and correspondence of the results for the prenatal screening program for erythrocyte immunisation, the tubes are made available for the HIP-study. For the HIP-study the platelet containing plasma of the stored blood tubes is serologically typed for HPA-1a, using a sandwich ELISA. In short, 20 µL of plasma containing platelets will automatically be pipetted into microtiter plates that have been coated with a monoclonal antibody CLBthromb/1 (C17) directed against glycoprotein IIIa, at a concentration of 3 µg/mL to capture all platelets from the plasma. Then HRP-conjugated B2G1, an antibody targeting HPA-1a, will be added and plates will be centrifuged and incubated for 45 minutes. Lastly, after washing of the plates, HRP-substrate solution will be added for 15 minutes and after stopping of this reaction the reactions will be quantified using an ELISA reader (Biochrom Anthos, Cambridge, United Kingdom). This HPA-1a ELISA was specifically designed for the HIP-study, thus for quick and high-throughput screening. All samples with an ELISA value below a defined optic density (OD) are called HPA-1a negative. The HPA-1a typing result is supported with an allelic discrimination polymerase chain reaction (PCR) assay. Plasma and buffy-coat of samples that are typed HPA-1a negative will be stored at -20°C, using only a study number. Additionally, for each HPA-1a negative case, material of one HPA-1a positive control will be stored simultaneously.

Because this first phase comprises serological HPA-1a typing, which is performed with fresh material, and a delay in the arrival of consent forms might exist, this phase is performed with all samples from pregnant women who did not decline participation for the HIP-study. All consecutive phases, such as antibody screening, risk-assessment development and clinical data retrieval, are solely performed in case of informed consent for the HIP-study.

Phase II. Of all samples stored with consent, obstetric caregivers will be contacted to obtain clinical information. An overview of these clinical parameters is provided in Figure 2. The clinical data will be stored in a secured digital database, designed by the LUMC, called ProMISe. First, study numbers of HPA-1a negative cases and HPA-1a positive controls with corresponding obstetric caregivers are entered into the database. Then, for each case, ProMISe randomly generates a code. Thereafter obstetric caregivers will receive a secured digital invitation to add clinical data to a digital case report form (CRF) for the cases from their practice. This secured invitation contains the initial personal data for the sample sent for the erythrocyte immunisation screening program together with the code generated by ProMISe. In the digital CRF they fill in this code and the clinical data. Clinical data is stored in ProMISe, only by anonymous study codes. This way, no personal information is being transferred or entered in our database, nor is the obstetric caregiver in possession of a key that links the anonymous study number

For peer review only

to personal information, nor does the caregiver know whether their patients or clients are HPA-1a negative or positive.

Phase III. The next step is to evaluate the incidence of alloimmunisation. Of all HPA-1a negative women that gave consent for the HIP-study, we will use the stored left-over plasma to screen for HPA-1a alloantibodies. For antibody screening the Pak Lx assay, a qualitative immunoassay, will be used, according to the manufacturer's' recommendations (LIFECODES Pak Lx Assay, Immucor GTI Diagnostics, Norcross, United States of America). In short, plasma samples are incubated with reconstituted beads and for the removal of unbound antibodies, the beads are washed. Next a conjugate (anti-human immunoglobulin G antibody conjugated to phycoerythrin) is added and incubated with the sample for 30 minutes at room temperature. Lastly, the Luminex 200 instrument is used to analyse the data. The advantage of this assay is that it is quick and uses only a small amount of plasma so there will be enough left-over for further testing in phase IV.

Phase IV. Combining the results from phase II and phase III will enable us to select cases of alloimmunisation with and without clinical manifestations of FNAIT to identify possible parameters to predict the development of (severe) bleeding complications. For this we will be testing different laboratory parameters as well as clinical parameters (Figure 2). Laboratory parameters that will be tested to assess risk at bleeding complications are: HLA-DRB3*0101 status, antibody level, Fc-core glycosylation and FcγRIII-binding index, endothelial cell binding, endothelial cell function. [24-28]

Sample size calculation

The HIP study was designed to assess the incidence of clinical relevant FNAIT in pregnant women in the Netherlands. Therefore, the incidence of ICH in HPA-1a immunised cases was compared with HPA-1a positive women. The estimated risk of ICH in immunised cases was 3%. For our power calculation we took a marge of 1% on the estimated incidence of ICH in FNAIT.[19],[29] In our control group we assumed a risk on symptomatic ICH of 4.9 in 10.000 (0.05%).[30] To achieve a power of 80% at an alpha level of 5%, we calculated that a total study population of 2,400 pregnant women is needed. Within this calculation, we took into account the unequal distribution between HPA-1a positive controls and immunised cases. We considered 5% of our total study population to consist of immunised cases, which means that we needed to include 120 immunised cases. Calculations were performed using logistic regression model making use of PASS 11.

Each year, approximately 60,000 RhD or Rhc negative pregnant women participate in the prenatal screening program for erythrocyte immunisation and are therefore eligible for enrolment in the HIP-study. To include 120 immunised cases, we need to include 1,200 HPA-1a negative women (immunisation rate of approximately 10%).[29] Because 2.1% of the Caucasian population is HPA-1a negative, the total study population should exist of 60,000 pregnant women (Table 1). Based on previous experience with the OPZI-study and the highly positive attitude toward potential HPA-screening in pregnancy women expressed in our previous study, the expected enrolment was 50%.[31, 32] This would correspond with a study period of two years.

Statistical analysis

Clinical data will be entered into a validated data capture system, provided and designed by the LUMC. The system is protected by password and contains internal quality checks to identify inaccurate or incomplete data. Laboratory data will be entered in a separate password protected database by

For peer review only

independent technicians, inaccessible to the researchers. Both clinical and laboratory data will be combined and further data management and analysis will be performed using SPSS (version 23.0) and Graphpad (version 8.0). An interim-analysis after one year will be performed.

Ethics and dissemination

The introduction of an antenatal screening program requires a careful balance between benefit and potential harm. To investigate the true natural history of FNAIT we aimed to collect data from pregnancies without additional interventions based on screening test results. This observational non-intervention design is ethically challenging. It would be unethical to share the antibody screening results with pregnant women but withhold them from therapy, therefore antibody screening will be performed far after due date. There will be no direct beneficial effect for pregnant women participating in the HIP study, pregnant women will be informed by their caregivers about this before they give consent to our study. Ethical approval for this study has been obtained from the Medical Ethical Committee Leiden-The Hague-Delft (METC-LDD) (P16.002).

Patient recruitment started in March 2017 and the study is planned to close to recruitment on the spring/summer of 2019. However, to ensure the inclusion of 1,000 – 1,500 HPA-1a negative women the inclusions period might take longer. Accurate predictions on the duration of the study will be made after interim-analysis at one year. Results will be published in relevant scientific journals and be disseminated in international conferences when inclusion and clinical data collection is finished.

Discussion

FNAIT can cause severe bleeding complications in fetuses and neonates, with a high risk of associated morbidity and mortality.(33) A preventive antenatal treatment, that effectively prevents these bleeding complications from occurring, is available.(5) In current practice, this prevention is only available in pregnancies with known alloimmunisation, usually after a previously affected child. To prevent these first cases as well, timely detection by prenatal and population-based screening is necessary.

Current lack of prospective non-interventional studies providing data on natural history of the disease as well as a reliable risk assessment tool to identify alloimmunised pregnancies that are at high risk for developing bleeding, complicates the implementation of such population-based screening. The aim of the HIP-study is to gather this missing knowledge necessary to adequately evaluate the potential efficacy and feasibility of prenatal population-based screening in order to timely detect and prevent FNAIT-related complications. With the current study design and logistics, making use of the current national screening program for red blood cell immunisation with a participation grade of 99.1%. We do not think that selection of RhD and Rhc negative women would influence the outcome of our study (i.e. immunization rate or bleeding symptoms). RhD and Rhc status has never been associated with platelet immunization during pregnancy and inheritance is unrelated since the RhD and Rhc genes are located on chromosome 1 and the HPA-1 allele on chromosome 17. Therefore we expect our results to give an adequate representation of the Dutch population of pregnant women.

A potential limitation of this study protocol is the lack of routine determination of neonatal platelet counts. However, the goals of potential screening and prevention of FNAIT is not to prevent a low platelet count as reflected as a laboratory result, but to prevent symptomatic disease, mainly ICH, with

For peer review only

associated morbidity caused by FNAIT. However, routine neonatal cerebral ultrasound is not performed either. Therefore, cases of subclinical ICH without symptoms (such as convulsions or reduced consciousness) or additional bleeding manifestations might be missed, although in theory these might lead to developmental problems later in life. However, major ICHs detected in prospective studies that did perform routine cerebral ultrasound, were cases that were symptomatic as well.[19, 34]

Further underestimation might occur due to the fact that we will perform only a single screening for anti-HPA-1a alloantibodies, that is at 27 weeks' gestation. Immunisations that occurs later in pregnancy or after delivery will not be detected. Also, immunisations that will result in complications and termination of pregnancy or IUFD before 27 weeks' gestation will not be identified. However, in terms of assessing feasibility and cost-effectiveness of population-based screening, a slight underestimation is unquestionably preferred to an overestimation. On the contrary we use another antibody screening method compared to earlier screening studies which is possibly more sensitive compared to the MAIPA technique (Monoclonal Antibody Immobilization of Platelet Antigens [35]) The PAK Lx assay was tested on a series with 100 cases with suspected FNAIT by our research group in 2014. In 26 of these cases anti-HPA-1a was detected by MAIPA, all cases were detected and one more by PAKLx. Overall, to our knowledge, the HIP-study will be a unique study to prospectively and observationally collect data on incidence and natural history of FNAIT by including this large number of pregnant women without performing any kind of intervention. Additionally, it will be the first study to be able to identify a unique and unbiased study group, that is, immunised pregnant women without disease and without intervention. This is the pre-eminent group to be used for the development of a risk-assessment platform in order to select immunised pregnancies that are at high risk to develop bleeding complications and would therefore benefit from antenatal preventive measures, such as IVIg treatment.

Contributorship statement

DW, DO, CES, MH designed the study, MK, LP, EL commented on the design of the HIP study; DW, TV, LP, DO, CES and MH wrote study materials and coordinated the study design, wrote and reviewed this paper.

Competing interests

The authors declare that they have no conflict of interest.

Funding

This project was funded by Landsteiner Foundation for Blood Transfusion Research (1440).

Data availability statement

A Data Availability Statement was not applicable, this article does not contain any data.

For peer review only

Reference list

1. Burrows RF, Kelton JG. Thrombocytopenia at delivery: a prospective survey of 6715 deliveries. Am J Obstet Gynecol. 1990;162(3):731-4.
2. Dreyfus M, Kaplan C, Verdy E, Schlegel N, Durand-Zaleski I, Tchernia G. Frequency of immune thrombocytopenia in newborns: a prospective study. Immune Thrombocytopenia Working Group. Blood. 1997;89(12):4402-6.
3. Winkelhorst D, Kamphuis MM, de Kloet LC, Zwaginga JJ, Oepkes D, Lopriore E. Severe bleeding complications other than intracranial hemorrhage in neonatal alloimmune thrombocytopenia: a case series and review of the literature. Transfusion. 2016.
4. Tiller H, Kamphuis MM, Flodmark O, Papadogiannakis N, David AL, Sainio S, et al. Fetal intracranial haemorrhages caused by fetal and neonatal alloimmune thrombocytopenia: an observational cohort study of 43 cases from an international multicentre registry. BMJ Open. 2013;3(3).
5. Winkelhorst D, Murphy MF, Greinacher A, Shehata N, Bakchoul T, Massey E, et al. Antenatal management in fetal and neonatal alloimmune thrombocytopenia: a systematic review. Blood. 2017;129(11):1538-47.
6. Mueller-Eckhardt C, Kiefel V, Grubert A, Kroll H, Weisheit M, Schmidt S, et al. 348 cases of suspected neonatal alloimmune thrombocytopenia. Lancet. 1989;1(8634):363-6.
7. Newman PJ, Derbes RS, Aster RH. The human platelet alloantigens, PlA1 and PlA2, are associated with a leucine33/proline33 amino acid polymorphism in membrane glycoprotein IIIa, and are distinguishable by DNA typing. J Clin Invest. 1989;83(5):1778-81.
8. Davoren A, Curtis BR, Aster RH, McFarland JG. Human platelet antigen-specific alloantibodies implicated in 1162 cases of neonatal alloimmune thrombocytopenia. Transfusion. 2004;44(8):1220-5.
9. Campbell S, Swann HR, Seif MW, Kimber SJ, Aplin JD. Cell adhesion molecules on the oocyte and preimplantation human embryo. Hum Reprod. 1995;10(6):1571-8.
10. Sajid M, Stouffer GA. The role of alpha(v)beta3 integrins in vascular healing. Thromb Haemost. 2002;87(2):187-93.
11. de Haas M, Thurik FF, Koelewijn JM, van der Schoot CE. Haemolytic disease of the fetus and newborn. Vox Sang. 2015;109(2):99-113.
12. Husebekk A, Killie MK, Kjeldsen-Kragh J, Skogen B. Is it time to implement HPA-1 screening in pregnancy? Curr Opin Hematol. 2009;16(6):497-502.
13. Kjeldsen-Kragh J, Ni H, Skogen B. Towards a prophylactic treatment of HPA-related foetal and neonatal alloimmune thrombocytopenia. Curr Opin Hematol. 2012;19(6):469-74.
14. Murphy MF, Williamson LM. Antenatal screening for fetomaternal alloimmune thrombocytopenia: an evaluation using the criteria of the UK National Screening Committee. Br J Haematol. 2000;111(3):726-32.
15. Blanchette VS, Chen L, de Friedberg ZS, Hogan VA, Trudel E, Decary F. Alloimmunization to the PlA1 platelet antigen: results of a prospective study. Br J Haematol. 1990;74(2):209-15.
16. Durand-Zaleski I, Schlegel N, Blum-Boisgard C, Uzan S, Dreyfus M, Kaplan C. Screening primiparous women and newborns for fetal/neonatal alloimmune thrombocytopenia: a prospective comparison of effectiveness and costs. Immune Thrombocytopenia Working Group. Am J Perinatol. 1996;13(7):423-31.
17. Davoren A, McParland P, Crowley J, Barnes A, Kelly G, Murphy WG. Antenatal screening for human platelet antigen-1a: results of a prospective study at a large maternity hospital in Ireland. BJOG. 2003;110(5):492-6.
18. Maslanka K, Guz K, Zupanska B. Antenatal screening of unselected pregnant women for HPA-1a antigen, antibody and alloimmune thrombocytopenia. Vox Sang. 2003;85(4):326-7.
19. Kjeldsen-Kragh J, Killie MK, Tomter G, Golebiowska E, Randen I, Hauge R, et al. A screening and intervention program aimed to reduce mortality and serious morbidity associated with severe neonatal alloimmune thrombocytopenia. Blood. 2007;110(3):833-9.
20. Zwiers C, van Kamp I, Oepkes D, Lopriore E. Intrauterine transfusion and non-invasive treatment options for hemolytic disease of the fetus and newborn - review on current management and outcome. Expert Rev Hematol. 2017;10(4):337-44.
21. van Gils PFT, L.; Hamberg-van Reenen, H.H.; van den Berg, M. Kosteneffectiviteit van preventie. Rijksinstituut voor Volksgezondheid en Milieu (RIVM). 2009.
22. Van der Linden A.M.A. SY, Oomen P., Vos K. Prenatale Screening Infectieziekten en Erytrocytenimmunisatie (PSIE). TNO/RIVM; 2018.
23. van der Ploeg K, Schönbeck, Y., Oomen, P., Vos, K. Prenatale Screening Infectieziekten en Erytrocytenimmunisatie (PSIE). 2018.
24. Kapur R, Kustiawan I, Vestrheim A, Koeleman CA, Visser R, Einarsdottir HK, et al. A prominent lack of IgG1-Fc fucosylation of platelet alloantibodies in pregnancy. Blood. 2014;123(4):471-80.
25. Kapur R, Heitink-Polle KM, Porcelijn L, Bentlage AE, Bruin MC, Visser R, et al. C-reactive protein enhances IgG-mediated phagocyte responses and thrombocytopenia. Blood. 2015;125(11):1793-802.
26. Sonneveld ME, Natunen S, Sainio S, Koeleman CA, Holst S, Dekkers G, et al. Glycosylation pattern of anti-platelet IgG is stable during pregnancy and predicts clinical outcome in alloimmune thrombocytopenia. Br J Haematol. 2016.
27. L'Abbe D, Tremblay L, Filion M, Busque L, Goldman M, Decary F, et al. Alloimmunization to platelet antigen HPA-1a (PIA1) is strongly associated with both HLA-DRB3*0101 and HLA-DQB1*0201. Hum Immunol. 1992;34(2):107-14.
28. Metcalfe P, Allen D, Chapman J, Ouwehand WH. Interlaboratory variation in the detection of clinically significant alloantibodies against human platelet alloantigens. Br J Haematol. 1997;97(1):204-7.
29. Kamphuis MM, Paridaans N, Porcelijn L, De Haas M, Van Der Schoot CE, Brand A, et al. Screening in pregnancy for fetal or neonatal alloimmune thrombocytopenia: systematic review. BJOG. 2010;117(11):1335-43.
30. Gupta SN, Kechli AM, Kanamalla US. Intracranial hemorrhage in term newborns: management and outcomes. Pediatric neurology. 2009;40(1):1-12.
31. Winkelhorst D, Loeff RM, van den Akker-Van Marle ME, de Haas M, Oepkes D. Women's attitude towards routine human platelet antigen-screening in pregnancy. Acta obstetricia et gynecologica Scandinavica. 2017;96(8):991-7.
32. Koelewijn JM, Vrijkotte TG, van der Schoot CE, Bonsel GJ, de Haas M. Effect of screening for red cell antibodies, other than anti-D, to detect hemolytic disease of the fetus and newborn: a population study in the Netherlands. Transfusion. 2008;48(5):941-52.
33. Winkelhorst D, Kamphuis MM, Steggerda SJ, Rijken M, Oepkes D, Lopriore E, et al. Perinatal Outcome and Long-Term Neurodevelopment after Intracranial Haemorrhage due to Fetal and Neonatal Alloimmune Thrombocytopenia. Fetal Diagn Ther. 2018:1-8.

For peer review only

34. Williamson LM, Hackett G, Rennie J, Palmer CR, Maciver C, Hadfield R, et al. The natural history of fetomaternal alloimmunization to the platelet-specific antigen HPA-1a (PlA1, Zwa) as determined by antenatal screening. Blood. 1998;92(7):2280-7.
35. Porcelijn L, Huiskes E, Comijs-van Osselen I, Chhatta A, Rathore V, Meyers M, et al. A new bead-based human platelet antigen antibodies detection assay versus the monoclonal antibody immobilization of platelet antigens assay. Transfusion. 2014;54(6):1486-92.

Tables

Table 1 – Estimated cases in HIP-study

	**%**	**Incidence**	**Cases in the Netherlands** *Total pregnancies n = 170,000*	**Cases during study period** *Total included n = 60,000**
HPA-1a negative	2.1	1 : 50	3,570	1,260
HPA-1a antibodies	10	1 : 400	428	126
Severe FNAIT	30	1 : 1,300	129	36
ICH	10 – 30	1 : 12,500	13	3-4

* Assuming 50% enrolment of the 60,000 RhD/Rhc negative women each year, for two years
FNAIT, fetal and neonatal alloimmune thrombocytopenia; HIP, HPA-screening in pregnancy; HPA, human platelet antigen; ICH, intracranial haemorrhage.

Figures

Figure 1 – Schedule of selection, enrolment and tests in the HIP-study
HPA, human platelet antigen; PSIE, prenatal screening of infectious diseases and erythrocyte immunisation; RhD, rhesus D; Rhc, rhesus c.

Figure 2 – Clinical parameters

These clinical parameters will be collected in the HIP study.

Supplemental information

Supplemental material 1 - Patient information leaflet

For peer review only

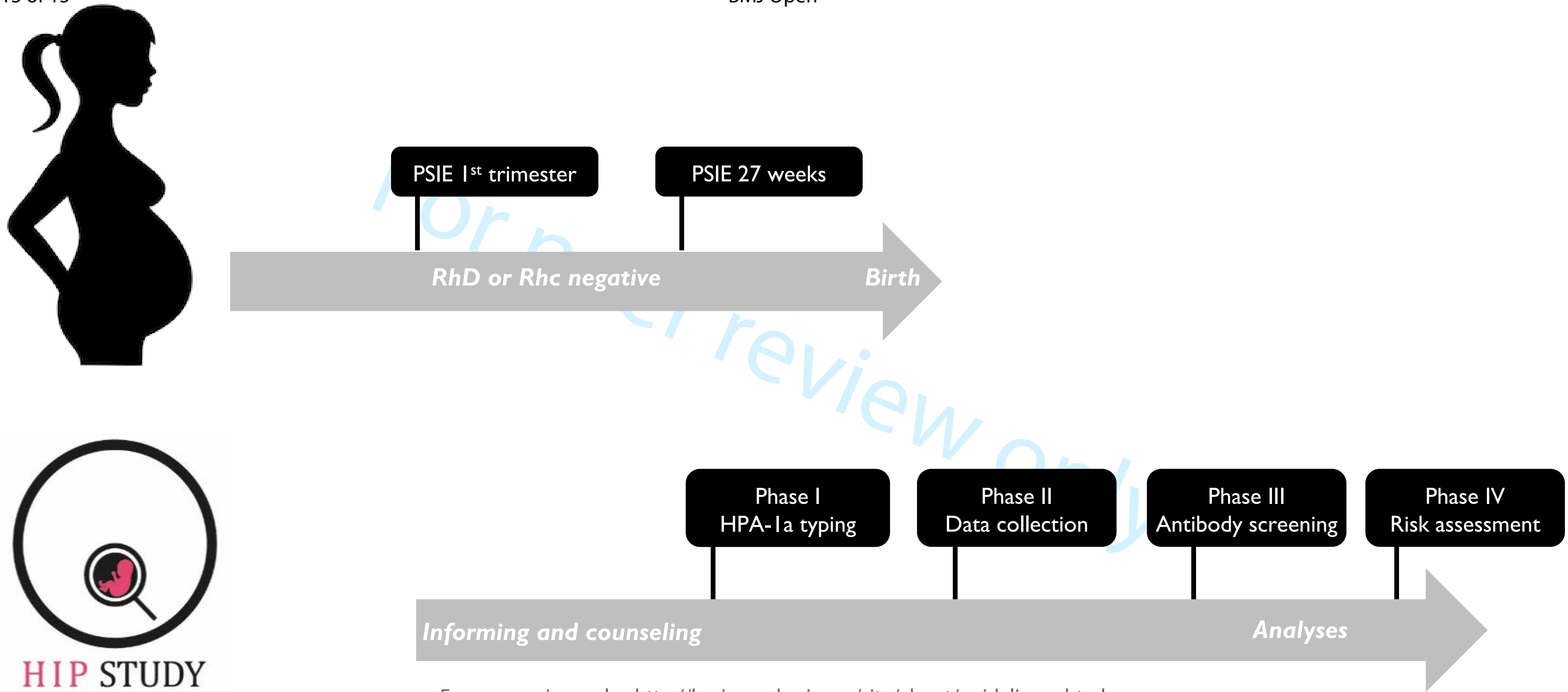

For peer review only - http://bmjopen.bmj.com/site/about/guidelines.xhtml

**Medical history:**	known with immune thrombocytopenic purpura (ITP)
**Obstetric history:**	previous pregnancies, deliveries, miscarriages (spontaneous as well as pregnancy terminations) or intra-uterine fetal demises.
**Pregnancy:**	gestational diabetes, hypertensive disorders (pregnancy-induced hypertension, pre-eclampsia), intrauterine growth restriction (IUGR).
**Perinatal:**	gestational age at delivery, mode of delivery, Apgar score, birth weight
**Neonatal:**	gender, chromosomal disorder, laboratory assessment (CRP or platelet count), consultation of pediatrician, admission to the neonatal care unit, mortality
**FNAIT-related:**	hematomas, petechiae, visceral bleeding, internal organ haemorrhage, intracranial haemorrhage, platelet count (if tested), treatment for thrombocytopenia

These clinical parameters will be collected in the HIP study.

415x190mm (96 x 96 DPI)

HIP-study

In the Netherlands all pregnant women are screened for antibodies against **red blood cells** as these antibodies can lead to anemia or jaundice in the newborn. In the blood of pregnant women, antibodies against **platelets** may also be present, these platelet antibodies can lead to bleeding problems in the newborn.

Can we prevent bleeding problems caused by antibodies against platelets? To answer this question we first need to know the following:

- How often are **antibodies** against platelets present in the blood of pregnant women?
- If these antibodies are present how often do they cause bleeding problems in newborn babies?
- Which newborns are at high risk of suffering from bleeding problems?
- Can we predict bleeding problems by testing the blood of pregnant women?
- How can we prevent these **bleeding problems** in newborn babies?

The HIP-study can provide answers to these questions. To answer all these questions , we need the help of midwives, gynaecologists and pregnant women. By agreeing to participate, you can make an important contribution to the HIP-study.

More information

For further information, please check
www.HIPstudie.nl
Or scan this QR-code to watch our informative video.

Please do not hesitate to consult your obstetric caregiver with any further questions you may have.
Or send an email to: **info@HIPstudie.nl**

Principal investigator:
drs. Dian Winkelhorst, medical doctor and researcher
Independent medical doctor:
Dr. M. Sueters, gynecologist, LUMC

Research study on platelet antibodies in pregnancy

HIP STUDY
HPA-screening In Pregnancy

Sanquin

LUMC
Leids Universitair Medisch Centrum

For peer review only - http://bmjopen.bmj.com/site/about/guidelines.xhtml

Why do you receive this flyer?

In the first trimester of your pregnancy, your red blood cell type was tested. The results showed that your red blood cells are RhD or Rhc negative. All RhD and Rhc negative pregnant women in the Netherlands are tested again at 27 weeks gestation by Sanquin in Amsterdam. We would like to perform an extra test, for the HIP-study, with the remainder of this blood sample. Here we ask your consent to perform this extra test.

What does this mean for you?

Participation is completely voluntary and will have no further consequences for the care you receive during your pregnancy. If you participate, no additional actions are necessary. No extra blood is drawn nor will you be personally contacted. You and your baby will be unaware of participation.

It is important to know that there is no personal gain in participating. However, your participation will contribute to improving knowledge on diseases caused by antibodies against platelets and will help to improve treatment during pregnancy in the future.

Which tests and actions are part of the HIP-study?

If you consent to participate in the HIP study, we will perform an extra test on the blood sample that has been already drawn. With this test we will determine the blood type of your platelets. This blood type is called **HPA-1a**. If you don't have this blood type, you are HPA-1a negative (1 in 50 women). If you are HPA-1a negative, we will store the tested blood sample. We will also store the blood sample from a small number of HPA-1a positive women as part of the so called 'control-group'.

After the expected delivery date all stored blood samples will be tested for antibodies against platelets . A member of the study team will contact your obstetric care giver to enquire on the health of your child during the first hours-days of life. All results and data will be anonymized and saved in a secured database. Results will not be reported to you or your obstetric care giver. The study results cannot be requested by third parties.

Participate?

Your obstetric care giver will ask you if you are willing to participate in the HIP-study. Your answer will be indicated on the HIP-study special check box on the blood collection form.

Background information

Our blood contains billions of cells. For example: red blood cells, white blood cells and **platelets**. All these cells express characteristics that are called blood types. Our body can produce **antibodies** against these blood types. During pregnancy a pregnant women can produce antibodies against the blood type of her child. Sometimes these antibodies are able to destroy the blood cells of the child. This can lead to disease and the need to start timely treatment.

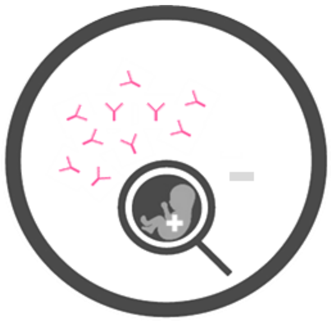

*'**HIP**' in **HIP**-study stands for:*
***H**PA-screening **I**n **P**regnancy*

***HPA** is an abbreviation for a blood type on platelets and means: Human Platelet Antigen.*

For peer review only - http://bmjopen.bmj.com/site/about/guidelines.xhtml
